# Polymyxins Bind to the Cell Surface of Unculturable *Acinetobacter baumannii* and Cause Unique Dependent Resistance

**DOI:** 10.1002/advs.202000704

**Published:** 2020-06-08

**Authors:** Yan Zhu, Jing Lu, Mei‐Ling Han, Xukai Jiang, Mohammad A. K. Azad, Nitin A. Patil, Yu‐Wei Lin, Jinxin Zhao, Yang Hu, Heidi H. Yu, Ke Chen, John D. Boyce, Rhys A. Dunstan, Trevor Lithgow, Christopher K. Barlow, Weifeng Li, Elena K. Schneider‐Futschik, Jiping Wang, Bin Gong, Bjorn Sommer, Darren J. Creek, Jing Fu, Lushan Wang, Falk Schreiber, Tony Velkov, Jian Li

**Affiliations:** ^1^ Infection & Immunity Program Biomedicine Discovery Institute and Department of Microbiology Monash University Melbourne 3800 Australia; ^2^ Monash Proteomics and Metabolomics Facility Monash University Melbourne 3800 Australia; ^3^ School of Physics and State Key Laboratory of Crystal Materials Shandong University Jinan 250100 China; ^4^ Department of Pharmacology and Therapeutics University of Melbourne Melbourne 3010 Australia; ^5^ School of Computer Science and Technology Shandong University Jinan 250100 China; ^6^ Department of Computer and Information Science University of Konstanz Konstanz 78457 Germany; ^7^ Drug Delivery, Disposition and Dynamics Monash Institute of Pharmaceutical Sciences Monash University Melbourne 3052 Australia; ^8^ Department of Mechanical and Aerospace Engineering Monash University Melbourne 3800 Australia; ^9^ State Key Laboratory of Microbial Technology Shandong University Qingdao Campus Qingdao 266237 China

**Keywords:** *Acinetobacter baumannii*, membrane lipidomics, molecular dynamics, polymyxin, polymyxin‐dependent resistance

## Abstract

Multidrug‐resistant *Acinetobacter baumannii* is a top‐priority pathogen globally and polymyxins are a last‐line therapy. Polymyxin dependence in *A. baumannii* (i.e., nonculturable on agar without polymyxins) is a unique and highly‐resistant phenotype with a significant potential to cause treatment failure in patients. The present study discovers that a polymyxin‐dependent *A. baumannii* strain possesses mutations in both *lpxC* (lipopolysaccharide biosynthesis) and *katG* (reactive oxygen species scavenging) genes. Correlative multiomics analyses show a significantly remodeled cell envelope and remarkably abundant phosphatidylglycerol in the outer membrane (OM). Molecular dynamics simulations and quantitative membrane lipidomics reveal that polymyxin‐dependent growth emerges only when the lipopolysaccharide‐deficient OM distinctively remodels with ≥ 35% phosphatidylglycerol, and with “patch” binding on the OM by the rigid polymyxin molecules containing strong intramolecular hydrogen bonding. Rather than damaging the OM, polymyxins bind to the phosphatidylglycerol‐rich OM and strengthen the membrane integrity, thereby protecting bacteria from external reactive oxygen species. Dependent growth is observed exclusively with polymyxin analogues, indicating a critical role of the specific amino acid sequence of polymyxins in forming unique structures for patch‐binding to bacterial OM. Polymyxin dependence is a novel antibiotic resistance mechanism and the current findings highlight the risk of ‘invisible’ polymyxin‐dependent isolates in the evolution of resistance.

## Graphical abstract

Herein, a mechanism of polymyxin‐dependent resistance in *Acinetobacter baumannii* is demonstrated. This unique dependence emerges only when i) the phosphatidylglycerol proportion in bacterial outer membrane increases to ≈35% and above, and ii) with ‘patch’ binding on the outer membrane by the rigid polymyxin molecules containing strong intramolecular hydrogen bonding, whereby polymyxin molecules enhance the membrane stability.

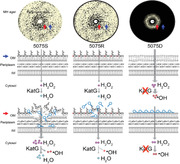

## 1 Introduction

The catalase‐positive, Gram‐negative opportunistic pathogen *Acinetobacter baumannii* causes life‐threatening nosocomial infections (e.g., pneumonia, sepsis, and meningitis) worldwide.^[^ ^1^ ^]^ *A. baumannii* infections are notoriously difficult to treat due to its extraordinary ability to evolve multidrug‐resistance (MDR).^[^ ^1^, ^2^ ^]^ Polymyxins (i.e., polymyxin B and colistin) are a group of cationic lipopeptide antibiotics and often used as a last‐line therapy against MDR Gram‐negative bacteria, including *A. baumannii*.^[^ ^3^, ^4^ ^]^ The exact mechanism of antibacterial killing by polymyxins remains unclear; however, the initial step involves electrostatic interactions between the l‐2,4‐diaminobutyric acid (Dab) groups of the polymyxin molecule and the 1‐ and 4′‐phosphate groups of lipid A in the bacterial outer membrane (OM).^[^ ^5^, ^6^ ^]^ Subsequently, the fatty acyl tails of polymyxins insert into the outer leaflet of the bacterial OM, resulting in membrane disorganization, cellular content leakage, and eventually cell death.^[^ ^5^, ^6^ ^]^ Alternative killing mechanisms of polymyxins include the inhibition of type II NADH‐quinone oxidoreductase and the generation of cytotoxic reactive oxygen species (ROS).^[^ ^7^, ^8^ ^]^ Resistance to polymyxins can emerge in *A. baumannii* during monotherapy and involves lipid A modifications with phosphoethanolamine (pEtN),^[^ ^9^ ^]^ or lipopolysaccharide (LPS) loss.^[^ ^10^, ^11^, ^12^, ^13^ ^]^ LPS loss causes significant OM remodeling, and is mediated by mutations in *lpxACD*, the first three genes of lipid A biosynthesis pathway.^[^ ^12^, ^13^ ^]^ Surprisingly, certain LPS‐deficient *A. baumannii* strains exhibit not merely a high‐level resistance to polymyxins, but also growth dependence on polymyxins, i.e., that bacteria only grow proximal to polymyxin‐containing discs on agar plates.^[^ ^14^ ^]^ However, not all the LPS‐loss strains are polymyxin dependent,^[^ ^12^, ^13^, ^14^ ^]^ indicating that LPS deficiency is insufficient for polymyxin dependence and other factors exist. Unfortunately, the current literature on the phospholipid composition of bacterial membranes in general is very sparse.

Polymyxin‐dependent resistance was reported only in *Acinetobacter* species thus far in the clinic.^[^ ^14^, ^15^, ^16^ ^]^ A recent clinical study of 45 patients undergoing colistin treatment showed that 40% *A. baumannii* isolates acquired colistin dependence and caused treatment failure; furthermore, after exposure to colistin in vitro, 32.9% of 149 colistin‐susceptible *A. baumannii* clinical isolates developed colistin‐dependent phenotype.^[^ ^17^ ^]^ Culturing *A. baumannii* in the presence of polymyxins is not routinely conducted in clinical microbiology diagnostic laboratories; therefore, polymyxin‐dependent resistant *A. baumannii* cannot be easily detected in the absence of polymyxins and represents a neglected and dangerous clinical issue. Here, we demonstrated that polymyxin‐dependent *A. baumannii* isolates were able to cause infection in mice. Using a model strain *A. baumannii* AB5075 (also a virulent MDR clinical isolate)^[^ ^18^ ^]^ and its polymyxin‐dependent mutant 5075D, our correlative systems pharmacological results and molecular dynamics simulations revealed that polymyxin‐dependent resistance in *A. baumannii* required at least four factors, LPS loss, distinctive phosphatidylglycerol (PG) rich OM, rigid polymyxin molecular structure, and polymyxin binding to the PG‐rich OM like ‘patches’.

## 2 Results

### 2.1 Characterization of Polymyxin Dependence

**Figure 1:**
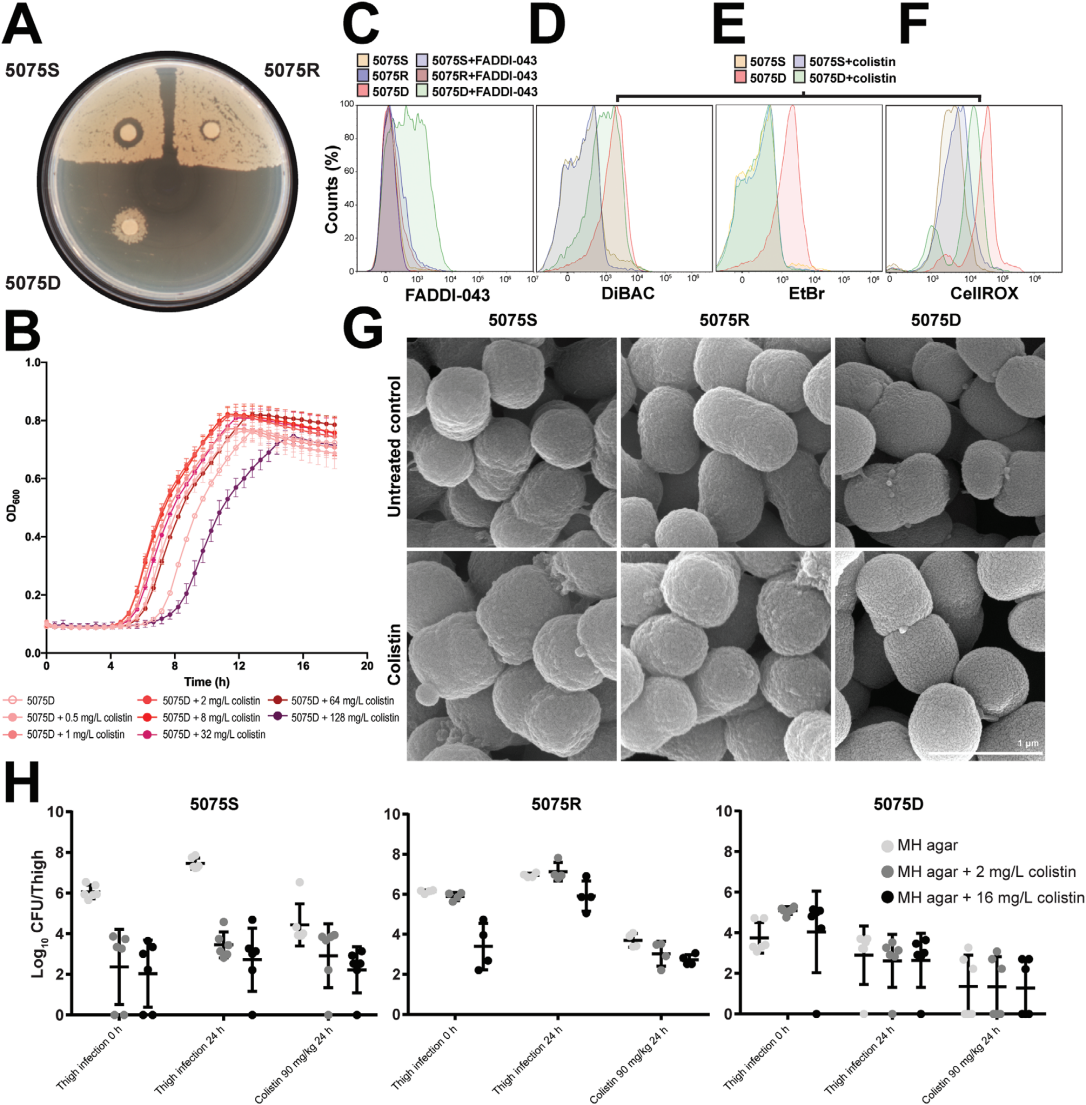
Phenotypic analyses of polymyxin‐dependent growth. A) 5075S, 5075R, and 5075D grown on MH agar with 3 µg colistin discs. Viable counting showed 1.9 × 10^6^ Colony Forming Unit (CFU) mL^−1^ cells for both 5075S and 5075R, and 3.3 × 10^5^ CFU mL^−1^ cells for 5075D. B) 5075D growth in MH broth with 0–128 mg L^−1^ colistin (*n* = 4). Data are shown as mean ± SD. C) Increased uptake of dansyl‐polymyxin probe FADDI‐043 in 5075D compared to 5075S and 5075R. D) Membrane depolarization of 5075D relative to 5075S with and without colistin treatment. DiBAC, bis‐(1,3‐dibutylbarbituric acid)trimethine oxonol. E) Metabolic activity of 5075D was lower than the wild‐type 5075S without colistin, but was restored in the presence of colistin. F) Enhanced oxidative stress in 5075D relative to 5075S with and without colistin treatment. G) SEM images of 5075S, 5075R, and 5075D in the absence and presence of colistin. H) In vivo bacterial burden of 5075S (*n* = 6), 5075R (*n* = 4), and 5075D (*n* = 6) with or without colistin treatment. Data are shown as mean ± SD.

A colistin‐dependent isolate 5075D was evolved from the wild‐type *A. baumannii* AB5075 (labeled as 5075S below, see the Experimental Section). Compared to colistin‐susceptible 5075S (minimum inhibitory concentration [MIC] = 0.25 mg L^−1^), strain 5075D was highly resistant to colistin (MIC > 128 mg L^−1^), but much more susceptible to rifampicin, meropenem, *β*‐lactams, and hydrogen peroxide (H_2_O_2_) (Table S1, Supporting Information). A colistin‐resistant mutant 5075R (MIC = 16 mg L^−1^) was also obtained, showing a similar susceptibility profile to other antibiotics as 5075S. When grown on Mueller–Hinton (MH) agar, 5075R showed a much smaller colistin inhibition zone than 5075S, while 5075D distinguished itself from normal polymyxin‐resistant phenotype by exhibiting a luxuriant growth around the disc containing 3 µg colistin (**Figure** **1**A). In MH broth, addition of colistin at concentrations ranging from 2 to 64 mg L^−1^ significantly improved the growth of 5075D (Figure 1B). Interestingly, addition of polymyxins slightly increased the resistance of 5075D to tetracycline. We measured the MICs of tetracycline in the absence and presence of polymyxin B nonapeptide (PMBN), respectively. Without PMBN, the MIC of tetracycline against 5075D was 0.0625 mg L^−1^; whereas with 2–64 mg L^−1^ PMBN, the MIC of tetracycline was increased to 0.125–0.25 mg L^−1^. FADDI‐043 is a dansyl‐polymyxin probe and was used for measuring polymyxin‐LPS binding interactions.^[^ ^19^ ^]^ Compared to 5075S and 5075R, much more FADDI‐043 bound to 5075D as shown by fluorescence‐activated cell sorting (FACS) (Figure 1C). Compared to 5075S, 5075D displayed more severe membrane depolarization (Figure 1D), reduced metabolic activity (Figure 1E), and higher oxidative stress in the absence of colistin (Figure 1F). Interestingly, addition of 3 mg L^−1^ colistin to the culture remarkably restored the metabolic activity and reduced the oxidative stress in 5075D (Figure 1E,F). Further examination using scanning electron microscopy (SEM) showed, after colistin treatment, blebbing cell surface in 5075S but no significant cell surface changes in 5075R or 5075D (Figure 1G). Despite attenuated virulence, 5075D was able to cause infection in the thighs of neutropenic mice and persisted after colistin treatment (90 mg kg^−1^) for at least 24 h (Figure 1H).

**Figure 2:**
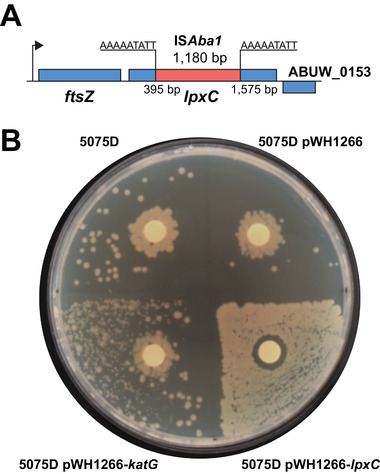
Insertional‐inactivated *lpxC* in 5075D and complementation. A) IS*Aba1* insertion in *lpxC* of 5075D. B) Disc diffusion assay of 5075D, 5075D with an empty vector, *katG* complementation, and *lpxC* complementation strains.

Whole genome sequencing and Sanger sequencing identified spontaneous mutations in 5075R and 5075D (Table S2, Supporting Information). Strain 5075R possessed 3 nonsynonymous substitutions (*pmrB* ^G315D^, ABUW_0507^A91P^, and *rpoB* ^F915L^), while 5075D had an integration of IS*Aba1* (1180‐bp in length) at nucleotide position 395 of *lpxC* (Figures S1 and 2A, Supporting Information) and 4 nonsynonymous substitutions (ABUW_0135^S25Stop^, *mrcA* ^M129T^, *katG* ^R609G^, and *rpoB* ^F915L^). These changes were present in 100% of reads, making the involvement of heterogeneity unlikely. ABUW_0135 encodes a putative signaling protein with unknown function, while *mrcA* encodes penicillin‐binding protein 1A and its inactivation could promote LPS loss in *A. baumannii*.^[^ ^20^ ^]^ The mutants with a single T26 transposon insertion in ABUW_0135, *mrcA*, *katG*, and *rpoB* were all susceptible to polymyxins (MIC ≤ 1 mg L^−1^, Tables S1 and S2, Supporting Information), indicating that the inactivation of these individual genes did not confer polymyxin‐dependent resistance. *lpxC* encodes UDP‐3‐*O*‐acyl‐*N*‐acetylglucosamine deacetylase that catalyzes the second reaction in lipid A biosynthesis.^[^ ^13^ ^]^ Our previous study showed that IS insertions in *lpxC* abolished its function, led to LPS loss and high‐level resistance to polymyxins.^[^ ^13^ ^]^ *katG* encodes a catalase and *A. baumannii* lacking *katG* showed increased susceptibility to H_2_O_2_.^[^ ^21^ ^]^ Interestingly, the complementation of *lpxC* completely eliminated polymyxin resistance and dependence, and restored the susceptibility, while the complementation of *katG* partially reduced polymyxin dependence (**Figure** **2**B). Taken together, the *lpxC* mutation was essential in the acquisition of polymyxin dependence in 5075D, while the *katG* mutation is not essential but played an important role here.

### 2.2 Integrative Analysis of Multiomics

To understand the mechanism of polymyxin dependence, correlative transcriptomics and metabolomics were conducted, and outer membrane proteomics was performed to support the transcriptomics results. Overall, the global gene expression and abundance of whole‐cell metabolites in 5075D were significantly different from those in 5075S and 5075R, and several interesting outer membrane proteins (OMPs) were evident in the proteomics results of 5075D; whereas colistin treatment resulted in profound changes in the metabolite and OMP abundance of 5075D. No common differentially expressed gene or altered metabolite was identified in 5075S, 5075R, and 5075D in response to 20 h colistin treatment (Figures S2A,B, S3A–C, and Datasets S1–S3, Supporting Information).

**Figure 3:**
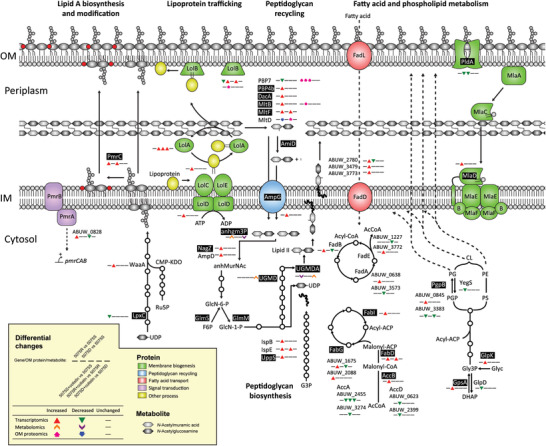
Transcriptomic, metabolomic, and OM proteomic analyses show the significantly perturbed membrane biogenesis by either genetic variations or colistin treatment. AcCoA, acetyl‐CoA; anhgm3p, *N*‐acetyl‐D‐glucosamine(anhydrous)*N*‐acetylmuramyl‐l‐Ala‐d‐Glu‐*meso*‐diaminopimelate; anhMurNAc, 1,6‐anhydro‐*N*‐acetylmuramic acid; CMP‐KDO, CMP‐3‐deoxy‐d‐manno‐octulosonate; DHAP, dihydroxyacetone phosphate; F6P, D‐fructose 6‐phosphate; G3P, glyceraldehyde 3‐phosphate; GlcN‐1‐P, D‐glucosamine 1‐phosphate; GlcN‐6‐P, D‐glucosamine 6‐phosphate; Glyc, glycerol; Glyc3P, glycerol 3‐phosphate; PGP, phosphatidylglycerol‐phosphate; PS, phosphatidylserine; UDCPDP, undecaprenyl diphosphate; UDP‐GlcNAc, UDP‐*N*‐acetyl‐d‐glucosamine; UGMD, UDP‐*N*‐acetylmuramoyl‐l‐Ala‐d‐Glu‐*meso*‐2,6‐diaminopimelate; UGMDA, UDP‐*N*‐acetylmuramoyl‐l‐Ala‐D‐Glu‐*meso*‐2,6‐diaminopimeloyl‐d‐Ala‐d‐Ala. The genes, metabolites, and proteins explicitly referred are shown in black background.

Polymyxin resistance in *A. baumannii* can be mediated by lipid A modification.^[^ ^10^, ^11^ ^]^ In 5075S, 20 h colistin treatment caused 2.3‐fold increase in the expression of lipid A phosphoethanolamine transferase gene *pmrC*. In the absence of colistin, the expression of *pmrC* was 3.2‐fold higher in 5075R than that in 5075S. Interestingly, without colistin treatment *lpxC* was downregulated by 2.6‐fold in 5075R relative to 5075S, but remained unchanged in 5075S and 5075R when exposed to colistin treatments (**Figure** **3**). Compared to untreated 5075S, several key genes related to the OM phospholipid biosynthesis and transport were differentially expressed in the untreated 5075D, including i) increased expression of *gpsA* (glycerol 3‐phosphate dehydrogenase, 1.8‐fold), *glpK* (glycerol kinase, 2.1‐fold), *mlaD* (OM lipid asymmetry maintenance protein, an component of the phospholipid retrograde transport system, 1.7‐fold) and *pgpB* (phosphatidylglycerophosphate phosphatase B, 1.7‐fold), and ii) decreased expression of *pldA* (phospholipase A, 2.0‐fold) (Figure 3). Metabolomics analysis showed significantly changed levels of phospholipids in untreated 5075D compared to untreated 5075S (Figure S3A,B, Supporting Information). Collectively, both transcriptomic and metabolomics results indicate increased phospholipid biosynthesis and perturbed phospholipid turnover in 5075D. Notably, compared to the untreated 5075S, the untreated 5075D significantly increased the expression of *accB* and *fabDGI* (Figure 3), as well as elevated the levels of fatty acids (Figure S3A,B, Supporting Information), indicating increased fatty acid biosynthesis for the substantial OM remodeling due to LPS loss. It should be noted that in 5075D, 20 h colistin treatment resulted in a remarkable increase (2.2‐ to 4.8‐fold) of 77 fatty acids and fatty acid derivatives, but no significant changes in the gene expression (Figure S3A–C and Datasets S1 and S2, Supporting Information).

The peptidoglycan recycling genes were exclusively upregulated in untreated 5075D compared to untreated 5075S (Figure 3). Consistently, the accumulation of *N*‐acetyl‐d‐glucosamine(anhydrous)*N*‐acetylmuramoyl‐l‐Ala‐d‐Glu‐*meso*‐2,6‐diaminopimelate and depletion of UDP‐*N*‐acetylmuramoyl‐l‐Ala‐d‐Glu‐*meso*‐2,6‐diaminopimeloyl‐d‐Ala‐d‐Ala were observed only in untreated 5075D, indicating an active peptidoglycan recycling and biosynthesis most likely due to the OM remodeling. Additionally, the expression of poly‐*β*‐1,6‐*N*‐acetylglucosamine biosynthesis and transport genes *pgaABCD* was significantly downregulated by 4.7‐ to 6.6‐fold in untreated 5075R and 5075D, compared to the untreated 5075S (Figure S4, Supporting Information).

A number of upregulated genes and depleted metabolites were exclusively observed in 5075D in the absence of colistin treatment, compared to 5075S, including those from tricarboxylic acid cycle, gluconeogenesis, energy generation, efflux pumps, and biosynthesis of nucleotides, arginine, methionine, and cysteine (Figure S4, Supporting Information). The expression of several ribosomal and aminoacyl‐tRNA synthetase genes was increased in untreated 5075D, compared to untreated 5075S, indicating an enhanced translation activity (Figure S4 and Dataset S1, Supporting Information). Compared to 5075S, 5075D was under a higher level of oxidative stress in the absence of colistin (Figure 1F). A number of genes associated with oxidative stress responses were differentially expressed in untreated 5075D compared to untreated 5075S, including the reduced expression of ferric uptake repressor *fur* and the increased expression of genes related to siderophore synthesis and transport, heme generation, glutathione biosynthesis, sulfate uptake and assimilation, and thiol/disulfide homeostasis maintenance (Figure S4, Supporting Information); all these results indicated a defense against aggravated oxidative stress in 5075D, which was due to the loss of LPS and KatG malfunction in particular.

### 2.3 Outer Membrane Remodeling

**Figure 4:**
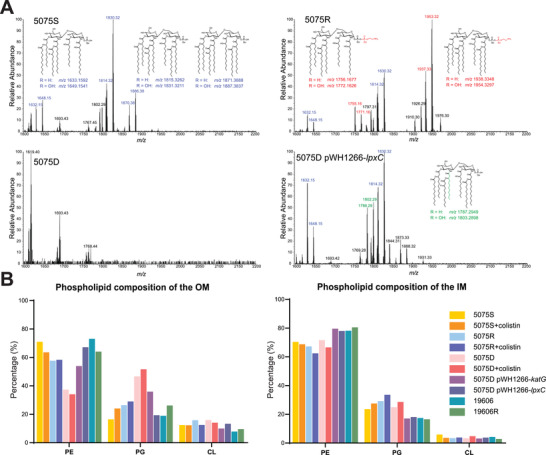
LPS depletion and significant changes of membrane phospholipids in 5075D. A) Lipid A profiles of 5075S, 5075R, 5075D, and 5075D pWH1266‐*lpxC* with putative lipid A structures. The *m*/*z* values of unmodified, modified, newly emerged unmodified (after *lpxC* complementation) lipid A are indicated in red, blue, and green, respectively. B) Membrane phospholipid compositions in different *A. baumannii* strains, including *katG* (5075D pWH1266‐*katG*) and *lpxC* (5075D pWH1266‐*lpxC*) complementation strains (*n* = 2). Data are shown as mean values.

The correlative multiomics results above indicated that the OM of 5075D was substantially remodeled due to LPS loss. Therefore, we conducted lipid A profiling and quantitative membrane lipidomics with 5075S, 5075R, and 5075D. Lipid A profiling showed that both 5075S and 5075R produced LPS, while LPS was absent in 5075D but restored by *lpxC* complementation (**Figure** **4**A). Specifically, both *hexa*‐acylated (*m*/*z* 1632.15 and 1648.15) and *hepta*‐acylated (*m*/*z* 1814.32, 1830.32, 1870.38, and 1886.38) lipid A species were present in 5075S (Figure 4A). After 20 h colistin treatment, a small proportion of pEtN modification at 1‐ or 4′‐phosphate was observed at *m*/*z* 1953.32 in 5075S (Figure S5, Supporting Information). In 5075R, the pEtN‐modified *hexa*‐ (*m*/*z* 1755.16 and 1771.16) and *hepta*‐acylated (*m*/*z* 1937.33 and 1953.32) lipid A species were dominant, regardless of colistin treatment (Figure 4A; and Figure S5, Supporting Information). In 5075D, lipid A was depleted (in the absence and presence of colistin) but restored by *lpxC* complementation (Figure 4A; and Figure S5, Supporting Information).

As LPS is predominant in the outer leaflet of Gram‐negative bacterial OM,^[^ ^22^ ^]^ its depletion causes significant OM remodeling with increased abundances of phospholipids and lipoproteins.^[^ ^23^, ^24^ ^]^ We therefore quantified the OM phospholipid abundances (Experimental Section, Supporting Information) and our results (Figure 4B; and Dataset S4, Supporting Information) demonstrated that phosphatidylethanolamine (PE) accounted for the majority (57.7–71.0%) of the total OM phospholipids in 5075S and 5075R, while its proportion was strikingly lower in 5075D (37.4% and 34.2% in the absence and presence of colistin, respectively) (Figure 4B; and Dataset S4, Supporting Information). Surprisingly, the PG content in OM phospholipids of 5075D was remarkably higher (46.7% and 51.7% in the absence and presence of colistin, respectively) than in 5075S and 5075R (16.5–29.1% with and without colistin, Figure 4B). Whereas the phospholipid composition of the IM remained largely unchanged in all three isolates regardless of colistin treatment (Figure 4B). Inactivation of PG biosynthesis genes PG phosphatase A (*pgpA*) or B (*pgpB*) significantly increased the susceptibility to colistin, which is indicated by drastically reduced viable cells of AB00256 (*pgpA*::T26, 6.6 × 10^2^ CFU mL^−1^) and AB08847 (*pgpB*::T26, 8.9 × 10^2^ CFU mL^−1^) compared to 5075S (2.1 × 10^10^ CFU/mL^−1^) after 4 mg L^−1^ colistin treatment for 24 h. These results showed a critical role of PG in developing colistin resistance in *A. baumannii*. Complementation of *katG* in 5075D resulted in significant increase of PE (up to 54.0% from 37.4% in untreated 5075D) and decrease of PG (down to 36.0% from 46.7% in untreated 5075D) in the OM, consistent with the partial loss of polymyxin dependence as shown in Figure 2B. This suggested that the OM remodeling in 5075D was partially induced by the oxidative stress due to the *katG* mutation. Whereas the complementation of *lpxC* restored the OM phospholipid composition similar to the levels in wild‐type 5075S with the proportions of PE and PG to 67.2% and 19.4%, respectively (Figure 4B). To test whether LPS deficiency would be sufficient to cause polymyxin dependence, we examined another LPS‐deficient, polymyxin‐resistant strain 19606R (colistin MIC > 128 mg L^−1^) derived from the wild‐type *A. baumannii* ATCC 19606 (colistin MIC = 0.5 mg L^−1^).^[^ ^12^ ^]^ Interestingly, 19606R showed no polymyxin dependence, and PE and PG in its OM accounted for 64.1% and 26.2%, respectively (Figure 4B). Taken together, these results showed, for the first time, that LPS loss causes polymyxin resistance, but not necessarily polymyxin dependence; while a high PG proportion in the LPS‐deficient OM is essential for polymyxin‐dependent resistance in *A. baumannii*.

### 2.4 Colistin Physically Stabilized 5075D OM

**Figure 5:**
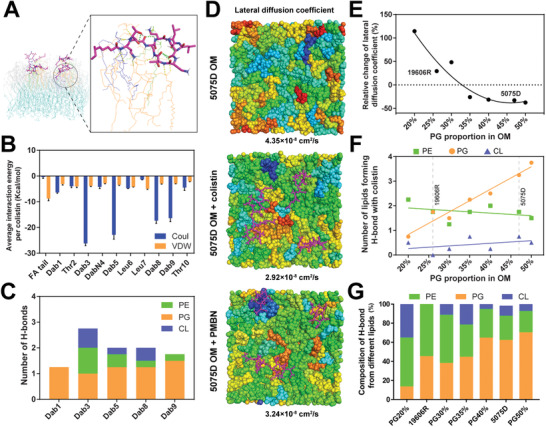
Molecular dynamics simulations show a patch binding of polymyxins to the OM of 5075D. A) Molecular interactions between colistin (violet) and phospholipid molecules in the outer leaflet of 5075D OM. The colistin‐interacting PG, PE, and CL are indicated in orange, green, and dark blue, respectively; while the noninteracting phospholipids in the outer and inner leaflets of OM are indicated in gray and light blue, respectively. The H‐bonds within colistin molecule and between colistin and phospholipid molecules are indicated by green and yellow dashed lines, respectively. B) The hydrophobic and electrostatic interaction energy of the fatty acyl tail and each amino acid residue of colistin. Interaction energy error bars were calculated using the block averaging method. C) The average number of H‐bonds between each colistin Dab residue and phospholipids. D) Binding of colistin or PMBN to the OM significantly reduced the overall membrane lateral diffusion coefficient in 5075D. Colistin and PMBN molecules are shown in violet. Color‐coding from blue to red indicates the phospholipids with low to high membrane lateral diffusion coefficient (LDC). E) Relative change of LDC with and without colistin at each specific PG proportion (20–50%), calculated by (LDC_colistin –_ LDC_control_)/LDC_control_ × 100%. Averaged LDC (*n* = 2 membranes) was calculated for 5075D. F) The averaged total number of phospholipids forming H‐bonds with colistin molecules at each specific PG proportion (20–50%). G) The composition of H‐bonds formed with different phospholipids at each specific proportion of PG (20–50%) in the OM.

Our multiomics results indicated that the unique OM composition played an essential role in polymyxin dependence; therefore, molecular dynamics simulations were conducted based upon our OM lipidomics results above (Figure 4B). Very interestingly, colistin uniquely bound to different phospholipids in the PG‐rich outer leaflet of the 5075D OM (**Figure** **5**A). The cyclic head of colistin molecule interacted with the surrounding phospholipid head groups in the LPS‐deficient 5075D OM in a patch binding manner (Movie S1, Supporting Information). On average, each colistin molecule interacted with 5.75 neighboring phospholipids and formed 12.25 hydrogen bonds (H‐bonds) therewith. For each colistin molecule, on average the five cationic Dab residues (i.e., Dab1, Dab3, Dab5, Dab8, and Dab9) interacted with the head groups of 3.40 PG, 1.20 PE, and 0.80 cardiolipin (CL), which contributed 69.7% of the total interaction energy (Figure 5B). Specifically, each colistin Dab residue formed 1.25–2.75 H‐bonds with phospholipids and Dab3 formed the maximum number of H‐bonds (Figure 5C). Among all the H‐bonds between colistin Dab residues and phospholipids in the outer leaflet of bacterial OM, 62.6% were formed with PG, even though PG only accounted for 46.7% of the OM phospholipids in 5075D (Figure 5C). Notably, Dab5, Dab8, and Dab9 were prone to form H‐bonds with PG; Dab1 formed H‐bonds only with PG; Dab3 formed H‐bonds equally with PE and PG (Figure 5C). Additionally, 5.0 H‐bonds were formed within a colistin molecule on average (Figure 5A). Upon colistin binding, the neighboring phospholipids were constrained, leading to limited movement of the phospholipids and a significantly declined lateral diffusion coefficient (from 4.35 × 10^−8^ cm^2^ s^−1^ to 2.92 × 10^−8^ cm^2^ s^−1^; Figure 5D), which supports the patch binding mode by colistin on the LPS‐deficient OM of 5075D. In 5075S OM, the lateral diffusion coefficient was 1.08 × 10^−8^ cm^2^ s^−1^. Further simulations with PMBN (derived by cleavage of the fatty acyl tail and Dab1 residue from polymyxin B) showed that PMBN also patch bound to 5075D OM and reduced the lateral diffusion coefficient to 3.24 × 10^−8^ cm^2^ s^−1^ (Figure 5D; and Movie S2, Supporting Information); these results showed that the polymyxin Dab residues (in particular Dab3, Dab5, Dab8, and Dab9) played major roles in membrane stabilization.

To test our hypothesis on the essentiality of unique PG composition in the OM for polymyxin dependence, we conducted a series of simulations with the OM PG composition varying from 20% to 50%. Excitingly, the results confirmed that colistin binding did not decrease membrane lateral diffusion until the PG content in the outer leaflet of the LPS‐deficient OM reached ≈35% and above (Figure 5E); and importantly, only when the PG composition was above this threshold, colistin molecules were able to uniquely patch bind to the OM via interacting more favorably with PG than PE and CL (Figure 5F,G). Therefore, LPS‐deficient strain 19606R with a low‐PG OM (26.2%, Figure 4B) showed only polymyxin resistance but no dependence. Collectively, our results reveal that a unique PG proportion in the outer leaflet of 5075D OM is essential to the patch binding of polymyxins via the Dab residues, which leads to the stabilization of the OM and polymyxin dependence.

### 2.5 A Rigid Molecular Structure of Polymyxins is Essential for the Dependent Resistance

It is unknown if the specific amino acid sequence and the ring structure of polymyxins or other polycationic peptides are essential for the dependent resistance. Therefore, we employed a chemical biology approach and further examined the dependence of strain 5075D in the presence of 7 different peptides: linear polymyxin B1, antimicrobial peptide LL‐37, polymyxin B1(Lys‐1, 3, 5, 8, 9), linear poly‐l‐arginine (RRRRRR), linear poly‐l‐lysine (KKKKKK), cyclic poly‐l‐arginine (RRRRRR), and cyclic poly‐l‐lysine (KKKKKK); the last two cyclic peptides represente a similar size of the polycationic ring as polymyxins. Notably, dependence was observed only in the presence of linear polymyxin B1 and polymyxin B1(Lys‐1, 3, 5, 8, 9), but not the other five peptides (Figure S6A, Supporting Information). Our results demonstrated that polymyxin dependence required a specific amino acid sequence, but not necessarily a cyclic structure.

Subsequently, molecular dynamics simulations were conducted to elucidate the mechanism of the interaction between 5075D OM and linear polymyxin B1 or linear poly‐l‐arginine. Similar to colistin and polymyxin B nonapeptide, linear polymyxin B1 significantly decreased the membrane lateral diffusion from 4.35 × 10^−8^ cm^2^ s^−1^ to 2.60 × 10^−8^ cm^2^ s^−1^ (Figure S6B, Supporting Information), suggesting that linear polymyxin B1 was also able to stabilize LPS‐deficient 5075D OM. Whereas the membrane lateral diffusion was increased from 4.35 × 10^−8^ cm^2^ s^−1^ to 5.58 × 10^−8^ cm^2^ s^−1^ (Figure S6C, Supporting Information) in the presence of poly‐l‐arginine, which is consistent with disc diffusion assay results (Figure S6A, Supporting Information). Notably, both linear polymyxin B1 and poly‐l‐arginine molecules could electrostatically interact with 6.5 membrane phospholipids (0.75 PE, 4.75 PG, and 1.00 CL for linear polymyxin B1; 2.00 PE, 3.25 PG, and 1.25 CL for poly‐l‐arginine) via 17.25 and 16.5 H‐bonds, respectively; however, the intramolecular electrostatic interactions were prominent in linear polymyxin B1 (4.75 H‐bonds formed per peptide molecule) than linear poly‐l‐arginine (1.0 H‐bonds formed per peptide molecule). Establishing a strong intramolecular polar interaction potentially increased the molecular rigidity of polymyxins during their interaction with bacterial OM, thereby stabilizing the LPS‐deficient OM and conferring polymyxin dependence.

## 3 Discussion

Polymyxin dependence is a unique resistance phenotype and has been overlooked in the clinic, as such isolates are nonculturable on conventional bacteriological agar plates in the absence of polymyxins. Antibiotic dependence has been reported in vancomycin‐resistant *Enterococcus*, linezolid‐resistant *Staphylococcus epidermidis*, and *β*‐lactam‐resistant *S. aureus*.^[^ ^25^, ^26^, ^27^ ^]^ However, these Gram‐positive bacteria either require antibiotic‐mediated gene induction to complement their functional defects,^[^ ^25^, ^27^, ^28^ ^]^ or display a functional adaptation to the antibiotic.^[^ ^26^ ^]^ In our preliminary experiments, we also explored if polymyxin dependence exists in other Gram‐negative bacteria (e.g., *Pseudomonas aeruginosa* and *Klebsiella pneumoniae*); however, no single polymyxin‐dependent strain could be selected in either species. As lipid A is essential for bacterial viability in *P. aeruginosa* and *K. pneumoniae*, lipid A modifications in polymyxin‐resistant strains do not cause dramatic OM remodeling, such as significant increase of PG component in the OM. Vancomycin‐resistant *Enterococcus* obligatorily requires continuous exposure to vancomycin for growth; intriguingly, polymyxin‐dependent *A. baumannii* can survive both in vitro and in vivo in the absence of polymyxins, even it is nonculturable on normal agar plates (Figure 1B–H). Indeed, clinical studies have shown a high rate of polymyxin dependence in polymyxin‐resistant *Acinetobacter* clinical isolates and higher rates of treatment failure in patients.^[^ ^17^ ^]^ Therefore, the probability of nosocomial transmission of polymyxin‐dependent resistant *A. baumannii* has been substantially under‐estimated.^[^ ^27^, ^28^ ^]^

Our results show that LPS loss is essential but only the first step of acquiring polymyxin‐dependent resistance. The significant changes related to the OM remodeling in 5075D (e.g., perturbed phospholipid biogenesis and increased fatty acid biosynthesis, lipoprotein transport, and peptidoglycan recycling; Figure 3) are consistent with previous transcriptomics results from LPS‐deficient *A. baumannii*.^[^ ^20^, ^29^ ^]^ The upregulated genes related to sulfur homeostasis and ferric utilization indicated severe oxidative stress in 5075D due to the *katG* mutation (Figure S4, Supporting Information). A very recent study discovered that the lytic transglycosylase gene *mltF* (ACICU_0 2898) could contribute to polymyxin dependence in *A. baumannii*.^[^ ^30^ ^]^ Its homologs (HMPREF0010_0 2077 in ATCC 19606, ABUW_0928 in AB5075) were also transcriptionally upregulated in untreated 19606R compared to untreated ATCC 19606,^[^ ^23^ ^]^ and in untreated 5075D compared to untreated 5075S; but remained unchanged in 5075D in the presence and absence of colistin (Figure 3). Collectively, these results indicate that *mltF* might be essential for the survival of LPS‐deficient *A. baumannii*, but not for polymyxin dependence.

Very limited information in previous literature suggested that polymyxin dependence was associated with the defects of lipid A biosynthesis;^[^ ^14^, ^17^, ^30^, ^31^ ^]^ however, not all LPS‐deficient *A. baumannii* isolates are polymyxin‐dependent (e.g., 19606R). A major finding of our study is that, besides the significantly remodeled OM due to LPS loss, the combination of another two factors is key for polymyxin dependence: i) a uniquely high proportion of PG in the OM (≥ 35%), and ii) cytotoxic ROS that was harmful due to the instability of the remodeled OM. Bacteria need meticulous regulation of their membrane phospholipid composition, in particular maintaining an appropriate ratio between zwitterionic phospholipids (e.g., PE) and anionic phospholipids (e.g., PG) to achieve an optimal membrane stability.^[^ ^32^ ^]^ In Gram‐negative bacterial OM, anionic PG normally accounts for < 30%.^[^ ^33^ ^]^ In polymyxin‐resistant, LPS‐deficient 19606R, the PG proportion in the OM was 26.2%; however, the PG proportion surprisingly increased to ≥35% in the polymyxin‐dependent *A. baumannii* strains (46.7% in 5075D and 36.0% in 5075D‐pWH1266:*katG*, see Figure 4). The proposed mechanism of polymyxin dependence was also discovered in at least another two *A. baumannii* strains FADDI‐AB018D (sequence type [ST] 2) and FADDI‐AB046D (ST1). Both strains exhibited deficiency of LPS due to loss‐of‐function mutations in lipid A biosynthesis genes (*lpxA* ^A141V^ for FADDI‐AB018D and *lpxC* ^G35Stop^ for FADDI‐AB046D), and a high molar proportion (>35%) of PG in the LPS‐deficient OM.^[^ ^34^ ^]^ To the best of our knowledge, this is the first set of quantitative membrane lipidomics data showing a high proportion of PG in the OM of Gram‐negative bacteria, which provides novel mechanistic information on the OM remodeling.

**Figure 6:**
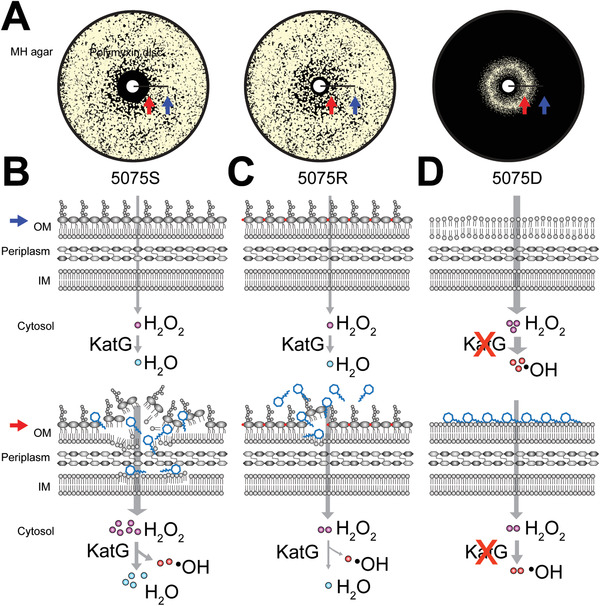
Unique surface patch binding of polymyxins stabilizes the remodeled LPS‐deficient PG‐rich OM and decreases ROS damage in 5075D, compared to 5075S and 5075R. A) 5075S, 5075R, and 5075D grown on MH agar with polymyxin discs. The distal and proximal positions with low and high concentrations of polymyxin are indicated by blue and red arrows, respectively. B) In polymyxin‐susceptible 5075S, external H_2_O_2_ molecules barely enter a cell and are scavenged by KatG catalase. Polymyxins disorganize the OM, significantly increase the influx of H_2_O_2_ which in turn saturates KatG. The extra H_2_O_2_ is converted to hydroxyl radical that produces mutagenic and lethal lesions. C) In polymyxin‐resistant 5075R, lipid A is modified with pEtN (red dots). Polymyxins cause less OM damage, and not many H_2_O_2_ molecules enter a cell which are scavenged. D) In polymyxin‐dependent 5075D, OM is unstable and leaky due to LPS loss and remodeling, and thus H_2_O_2_ molecules easily diffuse into a cell. Owing to KatG dysfunction, a large number of hydroxyl radicals are generated from H_2_O_2_. Polymyxins patch bind to the surface of remodeled, PG‐rich OM and stabilize the membrane, thereby decreasing the H_2_O_2_ influx and reducing the generation of hydroxyl radicals. The concentration of polymyxins in the distant area from the polymyxin disc (indicated by the blue arrows) was insufficient to stabilize the OM of 5075D; therefore, no bacterial growth was evident.

Our molecular dynamics simulation results showed that the PG‐rich OM of 5075D was much less stable; its lateral diffusion (4.35 × 10^−8^ cm^2^ s^−1^) was fourfold higher than that in 5075S (1.08 × 10^−8^ cm^2^ s^−1^) and 15‐fold higher than the reported value in *E. coli* (0.29 × 10^−8^ cm^2^ s^−1^).^[^ ^35^ ^]^ In addition, our FACS results showed a significantly reduced metabolic activity in 5075D compared to 5075S without colistin (Figure 1E). When the proportion of PG in the OM increased, the intermolecular electrostatic repulsion between the negatively‐charged PG head groups became profound and the positively‐charged molecules on membrane surface were able to stabilize its structure. Indeed, the surface patch binding of polymyxin “band‐aid” stabilized the remodeled LPS‐deficient PG‐rich OM (Figure 5D), thereby protecting bacterial cells from the ROS damage (Figures 1F and **6**) and restored metabolic activity (Figure 1E). As polymyxins bind to PG more favorably than PE (Figure 5C), such patch binding occurs only when the PG proportion in the OM is sufficiently high (≥ 35%, Figure 5E), wherein polymyxin molecules are able to form three or more H‐bonds with PG, a tripod‐like configuration to stabilize the LPS‐deficient OM (Figure 5F,G). The MD results of linear polymyxin B1 demonstrated that the rigid molecular structure via forming multiple intramolecular H‐bonds played a key role in stabilizing LPS‐deficient bacterial OM (Figure S6B,C, Supporting Information). In terms of the OM phospholipid composition, disruption of PG biosynthesis genes (*pgpAB*) caused significantly increased susceptibility to colistin (≈8‐log_10_ reduction of the number of viable cells compared to that in 5075S after 4 mg L^−1^ colistin treatment for 24 h). Our experimental and molecular dynamics simulation results (Figure 5E) also revealed that LPS‐deficient *A. baumannii* 19606R (a mutant derived from ATCC 19606) was only polymyxin‐resistant, but not dependent, as the PG content in its OM was too low to form sufficient phospholipid ‘rafts’ (26.2%, Figure 4B) enabling the surface patch binding of polymyxins. Consistently, our experimental results with the *katG* mutant and its complemented strain (5075D pWH1266‐*katG*) demonstrated that polymyxin dependence emerged (Figure 2B) in the *katG* mutant (PG = 36.0%, Figure 4B), which well supported our MD simulation results on the PG composition threshold for polymyxin dependence (≥35%). Furthermore, both experimental and molecular dynamics simulation results demonstrated that the fatty acyl chain of polymyxins was not essential for polymyxin dependence, as PMBN also stabilized the LPS‐deficient OM in 5075D. In summary, our results demonstrated that polymyxin dependence did not occur in LPS‐deficient *A. baumannii*, unless the PG proportion in the outer leaflet of the OM made the patch binding of polymyxins feasible (Figure 5E–G).

Collectively, our study discovered a novel antibiotic resistance mechanism, showing how polymyxins stabilize the LPS‐deficient OM of unculturable *A. baumannii* and lead to resistance to themselves. Our integrated multiomics results demonstrated, for the first time, the significantly increased proportion of PG in the OM of polymyxin‐dependent resistant *A. baumannii*. Polymyxin‐dependent resistance represents a novel structural and functional adaptation to antibiotic treatment in this top‐priority ‘superbug.’ The emergence of polymyxin‐dependent resistance is formidable and has significant clinical implications, considering that such dependent isolates are undetectable with current diagnostic procedures in hospitals and have caused treatment failure in patients. Our novel mechanistic findings may help optimize antibiotic therapies for life‐threatening infections caused by MDR *A. baumannii*.

## 4 Experimental Section

##### Bacterial Strains

*A. baumannii* strains were grown in MH media (Oxoid, UK) supplemented with antibiotics where appropriate. To obtain a polymyxin‐dependent mutant, 100 µL log‐phase bacterial culture and its serial dilutions were plated on MH agar containing 8 mg L^−1^ colistin (BetaPharma, China). Colonies were subsequently plated on MH agar with a sterile filter paper disc (8 mm in diameter) containing 3 µg colistin. Bacterial colonies that only grew near the disc but not on the rest of the plate were selected. Transposon insertion mutants were purchased from the Manoil Laboratory at the University of Washington, and cultured to test polymyxin susceptibility.^[^ ^36^ ^]^ *Escherichia coli* DH5*α* (Thermo Fisher Scientific, Waltham, MA) was grown in Luria‐Bertani broth supplemented with appropriate antibiotics. MICs of polymyxins were measured using broth microdilution methods according to Clinical and Laboratory Standards Institute standards.^[^ ^37^ ^]^ The susceptibility to other antibiotics was evaluated using VITEK 2 at Alfred Pathology Service (Alfred Hospital, Melbourne, Australia).

For complementation, fragments containing the full‐length genes and their native promoters were cloned to pWH1266. Strain 5075D was transformed with the constructs by electroporation (BioRad, Hercules, CA).^[^ ^38^ ^]^ Transformants were selected on MH agar with either 0.75 (*katG* complementation) or 5 µg mL^−1^ (*lpxC* complementation) tetracycline (Sigma‐Aldrich, USA); 0.75 µg mL^−1^ tetracycline was used for *katG* complementation because the transformant was LPS‐deficient and highly susceptible to tetracycline. The complementation was verified by Sanger sequencing at Monash Micromon (Clayton, Australia).

##### Phenotypic Assays

For disc diffusion assay, bacterial colonies were picked up from MH agar and resuspended in 0.9% w/w saline (McFarland = 1, i.e., 3 × 10^8^ CFU mL^−1^). MH agar plates were inoculated with 1:100 diluted culture using a swab. Sterile discs with 10 µg colistin (Beta Pharma, China), 10 µg polymyxin B (Beta Pharma, China), 10 µL 0.9% w/w saline, in‐house synthesized polymyxin analogues and peptides (15 µg polymyxin B nonapeptide, 15 µg linear polymyxin B1, 50 µg LL‐37, 15‐µg polymyxin B1(Lys‐1, 3, 5, 8, 9), 15 µg linear poly‐l‐arginine, 15 µg cyclic poly‐l‐arginine, 15 µg linear poly‐l‐lysine, and 15 µg cyclic poly‐l‐lysine) were placed onto agar plates which were incubated at 37 °C for 20 h. For growth curves,^[^ ^39^ ^]^ 1 µL of the above‐mentioned diluted culture was added to 99 µL fresh MH broth in the absence and presence of colistin (0.125, 8, and 64 mg L^−1^ for 5075S, 5075R, and 5075D, respectively) or/and catalase (20 U µL^−1^ for 5075D); real‐time optical density changes were recorded using a plate reader (Tecan, Switzerland). Strain 5075S and transposon insertion mutants AB00256 (*pgpA*::T26) and AB08847 (*pgpB*::T26) were cultured in cation‐adjusted MH broth (CaMHB) in the absence and presence of 4 mg L^−1^ colistin for 24 h with an inoculum of 10^6^ CFU mL^−1^. Samples were collected at 0 and 24 h and plated on nutrient agar (Oxoid) for viable counting.

##### Flow Cytometry Experiments

To investigate the cellular accumulation of polymyxins, early‐log bacterial MH cultures (OD_600_ 0.5) were treated with 3 mg L^−1^ fluorescent dansyl‐polymyxin probe FADDI‐043 for 1 h.^[^ ^19^ ^]^ MICs of FADDI‐043 for 5075S, 5075R, and 5075D were 2, 2, and 4 mg L^−1^, respectively. Cells were pelleted, washed twice and further diluted to 5 × 10^6^ CFU mL^−1^. Samples were then analyzed by flow cytometry (NovoCyte; ACEA Biosciences, USA) with excitation/emission at 405/530 nm. To check membrane polarity, metabolic activity, and cellular oxidative stress, bacteria grown at the edge of inhibition zone around a 3 µg colistin disc were collected. Specifically, bacterial cultures were directly diluted with saline to 5 × 10^6^ CFU mL^−1^, and then stained with 1.6 µg mL^−1^ bis‐(1,3‐dibutylbarbituric acid)trimethine oxonol [DiBAC_4_(3); Sigma‐Aldrich, St. Louis, MO] or 3.3 µg L^−1^ ethidium bromide (EtBr, Sigma‐Aldrich, St. Louis, MO) for 1 min. Samples were then analyzed by flow cytometry with excitation/emission at 488/530 nm [DiBAC_4_(3)] or 488/675 nm (EtBr). For oxidative stress, early‐log (OD_600_ = 0.5) cultures were treated with 3 mg L^−1^ colistin for 1 h with 1.0 × 10^−6^ m CellRox Green (Thermo Fisher Scientific).^[^ ^40^ ^]^ Bacterial cells were pelleted, washed once by saline, and resuspended. Samples were then diluted to 5 × 10^6^ CFU mL^−1^, injected to the flow cytometer and analyzed with excitation/emission at 488/530 nm.

##### Microscopy Imaging Experiments

Bacterial early‐log (OD_600_ = 0.5) cultures were treated with colistin (2 mg L^−1^ for 5075S and 10 mg L^−1^ for 5075R and 5075D) or saline (control) for 1 h, respectively. Samples were collected, fixed with 2.5% glutaraldehyde, dehydrated with gradient ethanol, dried with Leica EM CPD300 (Germany), and imaged with FEI TeneoVS 3D SEM (FEI, USA).^[^ ^41^ ^]^

##### Neutropenic Murine Thigh Infection Experiment

Animal experiments were approved by the Monash Animal Ethics Committee (animal ethics application approval number MARP/2018/011) and animals were maintained in accordance with the Australian Code of Practice for the Care and Use of Animals for Scientific Purposes. Eight‐week‐old, specific‐pathogen‐free, female Swiss mice (24–30 g) were obtained from Monash Animal Services (Clayton, Australia) and were fed, housed, and rendered neutropenic by the administration of cyclophosphamide.^[^ ^42^ ^]^ Colistin treatment (subcutaneous injection of colistin sulfate with 30 mg kg^−1^ every 8 h) was initiated at 2 h after the inoculation of 10^5^ CFU bacteria (i.e., 5075S, 5075R, and 5075D) in 50 µL saline. Bacterial burden in thighs was determined at 0 and 24 h after the treatment using MH agar containing 0, 2, and 16 mg L^−1^ colistin.^[^ ^42^ ^]^ Sanger sequencing was used to verify *lpxC* and *katG* mutations in samples of 5075D.

##### Multiomics Experiments

Bacterial cultures grown on Mueller–Hinton agar with a blank disc (i.e., no colistin) were used as untreated control samples; for treatment conditions, bacterial cultures were collected from the edge of the colistin inhibition zone. Bacterial samples were washed with saline for multiomics studies below. Genomic DNA and total RNA were extracted using DNeasy Blood and Tissue Kit and RNeasy Mini kit (QIAGEN, Hilden, Germany) and stored at −80 °C pending sequencing using Illumina MiSeq.^[^ ^9^ ^]^ Whole‐cell metabolomics samples were prepared using the previous method.^[^ ^43^ ^]^ For membrane lipidomics and proteomics, samples were prepared using sucrose gradient ultracentrifugation and total protein of each membrane fraction was analyzed by SDS‐PAGE with Coomassie brilliant blue staining.^[^ ^44^ ^]^ Prior to lipid extraction, membrane samples were normalized to 2 mg mL^−1^ by Pierce bicinchoninic acid (BCA) protein assay (Thermo Fisher Scientific). Membrane lipids were extracted by a double‐phase Bligh‐Dyer solution (Experimental Section, Supporting Information).^[^ ^45^ ^]^ The OM protein fraction was normalized to 200 µg with 100 mm TRIS buffer, followed by extraction and trypsin digestion. Lipid A was extracted using mild acid hydrolysis method.^[^ ^46^ ^]^ The processed samples for metabolomics, proteomics, membrane lipidomics, and lipid A profiling were analyzed by liquid chromatography—mass spectrometry (LC–MS) at Monash Proteomics and Metabolomics Facility (Clayton, Australia) (Experimental Section, Supporting Information).

##### Data Processing and Bioinformatic Analysis

Previous methods were employed for processing DNA/RNA‐Seq, lipidomics, and metabolomics raw data.^[^ ^46^, ^47^ ^]^ Differential expression analysis on the normalized count data was performed using the limma R package.^[^ ^48^ ^]^ Gene expression levels were considered significantly different across groups if the fold change (FC) was ≥ 2 and a false discovery rate (FDR)‐adjusted *P* value was ≤ 0.05. Genes with FC < 2 but FDR‐adjusted *P* ≤ 0.05 were also examined. Proteomics raw data were analyzed using MaxQuant to obtain protein identifications and their respective label‐free intensity.^[^ ^49^ ^]^ The intensity of metabolites and proteins was log_2_ transformed. Membrane phospholipids were quantified based on previous methods with calibration curves of PG, PE, and CL standards (Experimental Section, Supporting Information).^[^ ^50^ ^]^ The differentially abundant metabolites were identified using one‐way analysis of variance (ANOVA, two‐tail), with FC ≥ 2 and FDR‐adjusted *P* ≤ 0.05. Principal component analysis (PCA) was conducted in R.

##### Molecular Dynamics Simulations

All molecular dynamics simulations were implemented using GROMACS 5.1.2.^[^ ^51^ ^]^ The LPS‐depleted, symmetric OM was constructed by CELLmicrocosmos 2.2 MembraneEditor based on the membrane lipidomics results (Dataset S4, Supporting Information) with the reported phospholipids topology.^[^ ^52^, ^53^ ^]^ All membrane systems were solvated with SPC (simple point‐charge) water molecules, and the water molecules within the membrane bilayer were removed. The topology of colistin A and PMBN were created in PRODRG server.^[^ ^54^ ^]^ Molecular dynamics simulations were performed for i) 5075D OM only (*n *= 2), ii) 5075D OM with colistin (*n *= 2), iii) 5075D OM with PMBN (*n *= 2), iv) 19606R OM in the absence and presence of colistin (*n *= 1), v) LPS‐deficient OM with a varying PG composition (20–50%) in the absence and presence of colistin (*n *= 1), vi) 5075S OM (*n *= 1), vii) 5075D OM with linear polymyxin B1 (*n *= 1), and viii) 5075D OM with linear poly‐l‐arginine (*n *= 1) (Table S3, Supporting Information); each simulation required ≈40 h. In system v) the CL composition was fixed to 15%, while PE content varied along with PG to make the total percentage 100%; and in system vi) the lipid A accounted for 75% of the outer leaflet of OM. For each simulation, four polymyxin molecules were placed above the membrane surface at random positions. Energy minimization was first conducted to relieve unfavorable contacts in the system, followed by 1 ns equilibration in isothermal‐isovolumetric ensemble and 5 ns in isothermal‐isobaric ensemble; then a 100 ns production run was performed with the temperature maintained at 300 K using the Nose–hoover algorithm,^[^ ^55^ ^]^ and pressure maintained at 1 bar using semi‐isotropic pressure coupling with the Parrinello–Rhaman barostat.^[^ ^56^ ^]^ All simulations were conducted using the Australian National Computation Infrastructure and High‐Performance Computation Cluster at Shandong University (Jinan, China).

##### Statistical Analyses

Unless otherwise stated, all statistical analyses were performed using R with one‐way ANOVA (two‐tail) or limma package when appropriate.^[^ ^48^ ^]^ For ANOVA analyses, Fisher's least significant difference (LSD) post‐hoc tests were used. Benjamini–Hochberg procedure was used to adjust *P*‐values in multiple tests. Unless otherwise stated, all data show the mean and standard deviation (SD).

## Conflict of Interest

The authors declare no conflict of interest.

## Author Contributions

Y.Z., J.L., M.‐L.H., and X.J. contributed equally to this work. J.L. conceived and supervised the project. Y.Z. completed phenotypic assays, conducted DNA‐ and RNA‐Seq and wrote the manuscript. J.L. constructed the complemented strains, conducted SEM, and contributed to fluorescent imaging, lipid A profiling and lipidomics. M.‐L.H. conducted lipid A profiling, membrane lipidomics and metabolomics. X.J. conducted molecular dynamics simulations. M.A.K.A. conducted FACS experiments and N.A.P. synthesized the polymyxin analogues and peptides. Y.‐W.L. performed proteomics experiments. J.Z. prepared the bacterial culture for lipidomics and Y.H. prepared the bacterial culture for metabolomics. H.H.Y. isolated the polymyxin‐resistant and ‐dependent mutants. K.C. conducted in vivo experiments. J.W. contributed to animal work design. J.D.B contributed to molecular work design. R.A.D. and T.L. helped in membrane isolation. C.K.B. contributed to lipidomics data analysis and D.J.C. helped in metabolomics data analysis. W.L., B.S., F.S., B.G., and L.W. contributed to molecular dynamics simulations. E.K.S. contributed to SEM sample preparation and J.F. contributed to SEM data analysis. T.V. helped in antimicrobial susceptibility test. All authors contributed to manuscript revision.

## Supporting information

Supporting Information

Supplemental Movie 1

Supplemental Movie 2

Supplemental Excel 1

Supplemental Excel 2

Supplemental Excel 3

Supplemental Excel 4
