# Supplementary material for: Polymyxins Bind to the Cell Surface of Unculturable Acinetobacter baumannii and Cause Unique Dependent Resistance
Source: Adv Sci (Weinh). 2020 Jun 8;7(15):2000704. doi: 10.1002/advs.202000704 (PMC7403960; doi:10.1002/advs.202000704)
Supplement: Supplementary file 1 — Supporting Information [file ADVS-7-2000704-s001.pdf]

© 2020 Wiley-VCH GmbH

ADVANCED SCIENCE
Open Access

Supporting Information

for *Adv. Sci.,* DOI: 10.1002/advs.202000704

Polymyxins bind to the cell surface of unculturable Acinetobacter baumannii and cause unique dependent resistance

*Yan Zhu#, Jing Lu#, Mei-Ling Han#, Xukai Jiang#, Mohammad A. K. Azad, Nitin A. Patil, Yu-Wei Lin, Jinxin Zhao, Yang Hu, Heidi H. Yu, Ke Chen, John D. Boyce, Rhys A. Dunstan, Trevor Lithgow, Christopher K. Barlow, Weifeng Li, Elena K. Schneider-Futschik, Jiping Wang, Bin Gong, Bjorn Sommer, Darren J. Creek, Jing Fu, Lushan Wang, Falk Schreiber, Tony Velkov, Jian Li**

Supporting Information

Polymyxins bind to the cell surface of unculturable *Acinetobacter baumannii* and cause unique dependent resistance

Yan Zhu#, Jing Lu#, Mei-Ling Han#, Xukai Jiang#, Mohammad A. K. Azad, Nitin A. Patil, Yu-Wei Lin, Jinxin Zhao, Yang Hu, Heidi H. Yu, Ke Chen, John D. Boyce, Rhys A. Dunstan, Trevor Lithgow, Christopher K. Barlow, Weifeng Li, Elena K. Schneider-Futschik, Jiping Wang, Bin Gong, Bjorn Sommer, Darren J. Creek, Jing Fu, Lushan Wang, Falk Schreiber, Tony Velkov, Jian Li.*

***Correspondence to**: Professor Jian Li at 19 Innovation Walk, Monash University, Clayton campus, Melbourne 3800, Australia. Telephone: +61 3 9903 9702. Facsimile: + 61 3 9905 6450.

Email: jian.li@monash.edu

#Equal contribution.

This PDF file includes:

SI Appendix

Figures S1 to S6

Tables S1 to S3

Legends for Movies S1 to S2

Legends for Datasets S1 to S4

SI References

Other supplementary materials for this manuscript include the following:

Movies S1 to S2

Datasets S1 to S4

SI Appendix

Methods

Purification of the outer and inner membranes. Samples of bacterial outer and inner membranes were prepared as described previously. Briefly, bacterial cells were collected from 400 mL normalized cultures (OD_{600} = 0.8~1.0, ~2×10^8 CFU/mL; 5,000 × *g*, 10 min, 4°C), and washed with 200 mL of 10 mM Tris-HCl (pH 7.5). Cells were then resuspended in 5 mL of 10 mM Tris-HCl containing 0.75 mM sucrose (pH 7.5). Lysozyme (50 µg/mL) and phenylmethylsulfonyl fluoride (2 mM) were added to the suspension, followed by the addition of 10 mL of 1.65 mM EDTA (pH 7.5) before cell homogenization using EmulsiFlex (Avestin, Canada) at 15,000 psi. Total membranes were then collected by ultracentrifugation with a Ti 70.1 rotor using a Beckman Coulter Optima L-90K ultracentrifuge (132,000 × *g*, 45 min, 4°C) and resuspended in 25% (w/v) sucrose (5 mM EDTA). The inner and outer membranes were separated by a six-step sucrose gradient (35%:40%:45%:50%:55%:60%, w/v, sucrose in 5 mM EDTA, pH 7.5) through ultracentrifugation with a SW40 Ti rotor (205,000 × *g*, 17 h, 4°C). Membrane fractions (1 mL of each) were collected using a fractionator (Teledyne ISCO, USA) with 70% (w/v) sucrose in 5 mM EDTA (pH 7.5) as displacing fluid, combined according to SDS-PAGE with coomassie brilliant blue staining, and stored at -80°C.

Preparation of OM proteomics samples. The OM protein fraction samples were normalized to 200 µg with 100 mM Tris buffer, followed by protein reduction for 10 min at 95°C with 10 mM tris(2-carboxyethyl)phosphine (Sigma-Aldrich) and 40 mM chloroacetamide (Sigma-Aldrich). The resulting samples were added to an ice-cold chloroform/methanol/water mixture (2:1:1.5, v/v) and incubated on ice for 10 min followed by centrifugation at 13,000 × *g* for phase separation. Aqueous phases were discarded and the organic phases were dried using a Speedvac concentrator (Thermo Fisher Scientific). Total protein extract was subsequently reconstituted with 50 mM triethylammonium bicarbonate buffer (Sigma-Aldrich). Finally,

trypsin (1:100, w/w) was added to each sample and incubated for 16 h at 37°C with gentle agitation. After digestion, samples were treated with formic acid (1:100, v/v) to stop the trypsin activity and the resulting peptides were extracted using Pierce OMIX C18 ZipTips (Thermo Fisher Scientific) following the manufacturer's instruction. All chemicals were purchased from Sigma Aldrich (St. Louis, MO, USA).

Preparation of membrane lipid samples. The above membrane samples were normalized to 2.00 mg/mL based on a bicinchoninic acid (BCA) assay.[1] Membrane lipids were extracted with a double-phase Bligh-Dyer solution (chloroform/methanol/water, 1:1:0.9, v/v).[2] The lower phase was collected, dried under nitrogen gas stream, and reconstituted in 150 μL butanol/methanol (1:1, v/v). Particle-free samples (100 μL) were collected in LC-MS vials after centrifugation (14,000 × *g*, 10 min at 4°C) and stored at -20°C prior to LC-MS analysis. Notably, 20 μL of each membrane lipid sample was combined to prepare a pooled quality control (PQC) sample. Specifically, 400 μL PQC was dried under nitrogen gas stream and reconstituted in 40 μL butanol/methanol (1:1, v/v) to generate a concentrated sample for lipid identification. Commercial 17:0-14:1(9Z) PE (Catalogue # 1104), 17:0-14:1(9Z) PG (Catalogue # 1204) and $(15:0)_3$-16:1 CL (Catalogue # 1803) from Avanti Lipids were mixed and diluted with the above concentrated PQC to make phospholipid standards at 0.1, 0.25, 0.5, 1.0, 2.5 and 5.0 μM.

Preparation of lipid A samples. Lipid A was extracted using mild acid hydrolysis with slight modifications.[3] Briefly, cells were pelleted from 100 mL normalized culture (OD_{600} = 0.50, ~10^8 CFU/mL; 3,220 × *g*, 20 min) and washed twice with 5 mL phosphate-buffered saline (PBS). Cell pellets were then resuspended in 19 mL single-phase Bligh-Dyer solution (chloroform/methanol/water, 1:2:0.8, v/v), pelleted (3,220 × *g*, 15 min), washed once with 5 mL single-phase Bligh-Dyer solution, and resuspended in 10.8 mL 50 mM sodium acetate (pH 4.5). Samples were sonicated with a constant duty cycle (20 s at 50% output) using a probe tip

sonicator (Misonix, USA) and then incubated in a boiling water bath for 45 min. To extract lipid A, 12 mL chloroform and 12 mL methanol were added to the 10.8 mL cooled hydrolysis solution to make a double-phase Bligh-Dyer solution (chloroform/methanol/water, 1:1:0.9, v/v). The lower phase containing lipid A was collected and dried under nitrogen gas stream.

Liquid chromatography – mass spectrometry analyses. The metabolomics, membrane lipidomics and lipid A profiling were conducted on a Dionex UltiMate 3000 high-performance liquid chromatography (HPLC) in tandem with a Q-Exactive Orbitrap mass spectrometer (Thermo Fisher Scientific, Waltham, MA, USA). The electrospray voltage was set as 3.50 kV, and nitrogen was used as collision gas. For each assay, all samples were randomized and analyzed in a single LC-MS batch to minimize any potential variation.

Metabolomics samples were analyzed by a full MS scan with a mass range from 85 to 1,275 *m/z* in both positive and negative mode at a resolution of 35,000. A ZIC-pHILIC column (5 µm, polymeric, 150 × 4.6 mm; SeQuant, Merck) was maintained at 25°C. The LC solvent consisted of 20 mM ammonium carbonate (A) and acetonitrile (B) with a multi-step gradient system from 80% B to 50% B over 15 min, then to 5% B at 18 min, followed by a wash with 5% B for another 3 min, and re-equilibration for 8 min with 80% B at a flow rate of 0.3 mL/min. Chromatographic peaks, signal reproducibility and analyte stability were monitored by the assessment of pooled quality control PQC samples (10 µL of each sample) analyzed periodically throughout the batch, internal standards and total ion chromatograms for each sample. Mixtures of pure standards containing over 300 metabolites were analyzed within the batch in aid of metabolite identification.

A Dionex UltiMate 3000 RSLCnano system equipped with a Dionex UltiMate 3000 RS autosampler, an Acclaim PepMap RSLC analytical column (75 µm × 50 cm, nanoViper, C18, 2 µm, 100Å; Thermo Fisher Scientific) and an Acclaim PepMap 100 trap column (100 µm × 2 cm, nanoViper, C18, 5 µm, 100Å; Thermo Fisher Scientific) was employed in the

membrane proteomics study. Tryptic peptides were separated by increasing concentrations of 80% acetonitrile/0.1% formic acid at a flow of 250 nL/min for 128 min and analyzed with a QExactive Plus mass spectrometer (Thermo Fisher Scientific) operated in data-dependent acquisition mode using in-house, LFQ-optimized parameters.

Specifically, the eluent was nebulized and ionized using a nano electrospray source (ThermoFisher Scientific) with a distal coated fused silica emitter (New Objective). The capillary voltage was set at 1.7 kV and the Q Exactive mass spectrometer was operated in the data dependent acquisition mode to automatically switch between full MS scans and subsequent MS/MS acquisitions. Survey full scan MS spectra (*m/z* 375-1,800) were acquired in the Orbitrap with 70,000 resolution (at *m/z* 200) after accumulation of ions to a 3×10^6 target value with a maximum injection time of 30 ms. Dynamic exclusion was set to 20 s. The 10 most intense multiply charged ions ($z \geq 2$) were sequentially isolated and fragmented in the collision cell by higher-energy collisional dissociation (HCD) with a fixed injection time of 60 ms, 17,500 resolution and automatic gain control (AGC) target of 5×10^4.

The membrane lipid samples were analyzed by full MS scan (with a mass range from 140 to 2,000 *m/z* and a resolution at 70,000) and MS/MS scan (with a mass range from 450 to 1,300 *m/z* and a resolution at 17,500 in both positive and negative mode). The above concentrated PQC samples were analyzed by MS/MS scan with a resolution at 17,500 and a mass range from 450 to 2,000 *m/z* within the same batch. The samples were maintained at 4°C and eluted by the above-mentioned mobile phases through an Ascentis Express C8 column (50 × 4.6 mm, 2.7 μm; Sigma-Aldrich). The linear gradient started from 100% mobile phase A to a final composition of 35% mobile phase A and 65% mobile phase B over 24 min at a flow rate of 0.2 mL/min. Linear regression with $1/x$ weighting for the phospholipid standards (Supplementary Figure 6) which were analyzed within the same batch was used to create the standard calibration curve. PQC samples containing standard PG, PE and CL at 0.3, 0.8 and

4.0 μM were employed to assess the accuracy and reproducibility of calibration curves (Supplementary Figure 6).

Structural and semi-quantitative analysis of lipid A were conducted in negative mode with a mass range from 167 to 2,500 *m/z* and a resolution at 70,000. A Synergi Hydro-RP 80 Å column (50 × 2 mm, 4 μm; Phenomenex, USA) was maintained at 40°C, while the samples were stored at 4°C. Lipid A samples dissolved in 200 μL chloroform/methanol/isopropanol/water (1:1:1:0.5, v/v) were eluted using a gradient that consisted of mobile phase A (40% isopropanol and 60% Milli-Q water with 8 mM ammonium formate and 2 mM formic acid) and mobile phase B (98% isopropanol and 2% Milli-Q water with 8 mM ammonium formate and 2 mM formic acid). The flow rate was 0.2 mL/min within the first 15 min and increased to 0.5 mL/min from 16 to 22 min. The gradient started with 70% mobile phase A and 30% mobile phase B, followed by a linear gradient to a final composition of 100% mobile phase B, which was maintained for 4 min. A 3-min re-equilibration of the column with 70% mobile phase A was performed prior to the next injection.

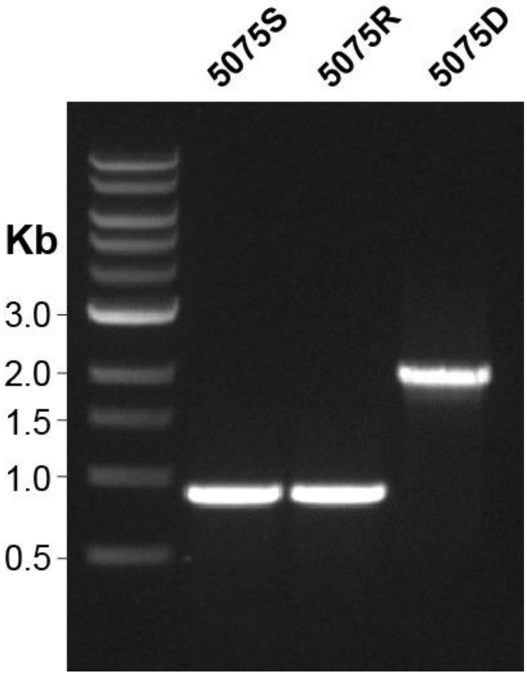

Figure S1. Electrophoresis of *lpxC* amplification from 5075S, 5075R and 5075D.

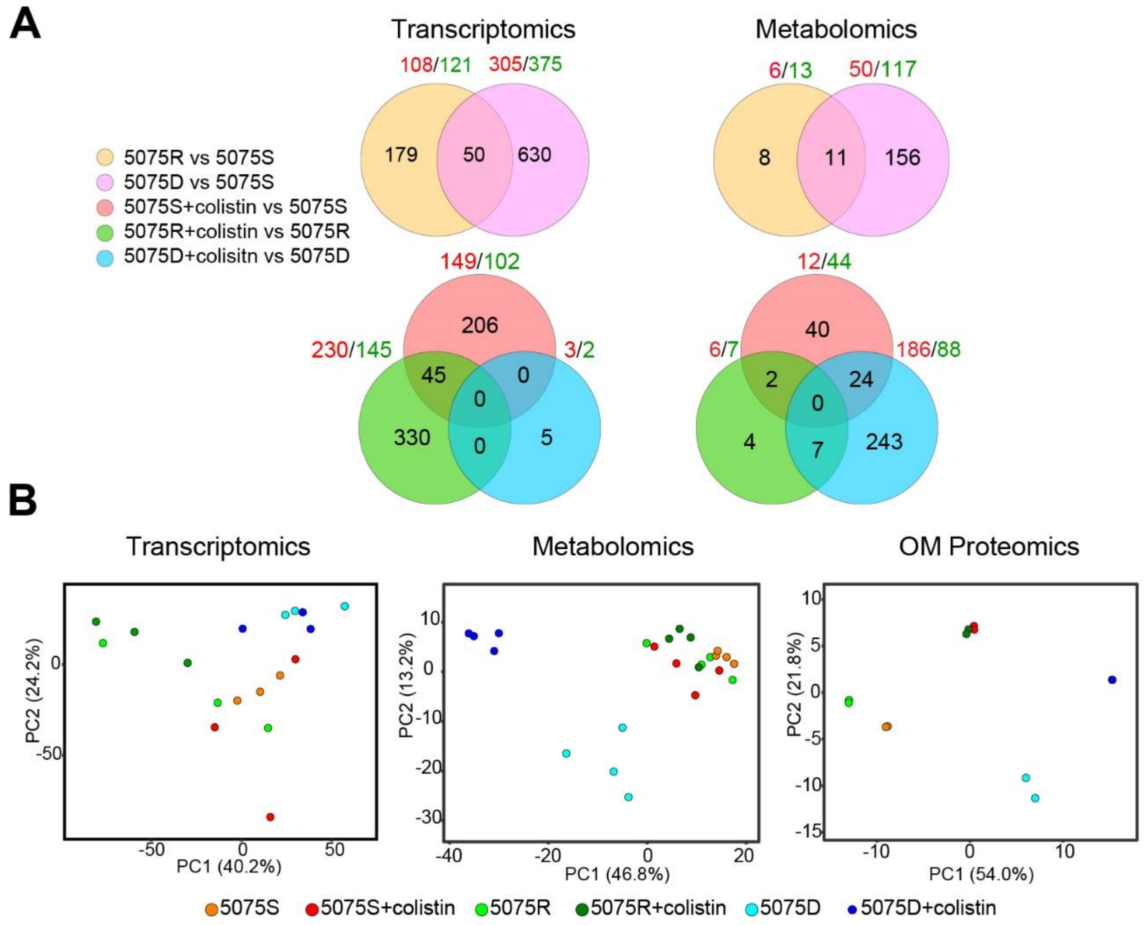

Figure S2. Significant changes in the transcriptome, metabolome and OM proteome of *A. baumannii* strains due to genetic variations and colistin treatment. A) Numbers of genes and metabolites significantly different (FC ≥ 2, FDR-adjusted $P \leq 0.05$) in multiple comparisons, with red/green numbers indicating significantly increased/decreased gene expression and metabolite level, respectively. B) PCA plots for transcriptomics, metabolomics and OM proteomics data.

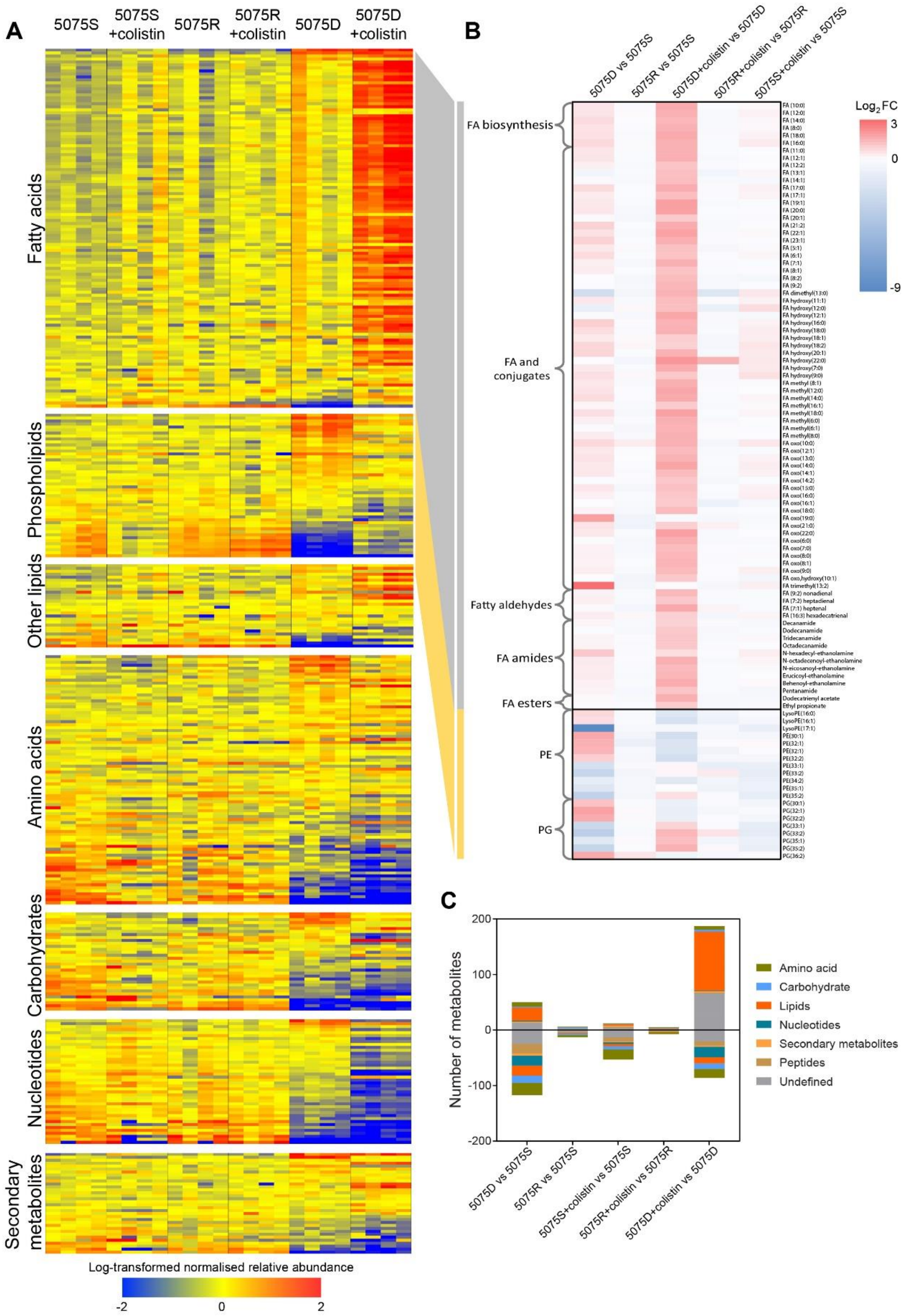

Figure S3. Significant metabolic changes in 5075D. A) Metabolite abundance in 5075S, 5075R and 5075D in the absence and presence of colistin treatment (*n* = 4). Data are shown as log-transformed normalized relative abundance. B) Putatively identified fatty acids, fatty acid

derivatives and phospholipids that were significantly perturbed (one-way ANOVA, two-tail, Fisher's LSD post-hoc test, FC ≥ 2, FDR-adjusted $P \leq 0.05$) in untreated 5075D compared to untreated 5075S, and in colistin-treated 5075D compared to untreated 5075D. Data are shown as $\log_2$-transformed fold changes. C) Numbers of differentially abundant (one-way ANOVA, two-tail, Fisher's LSD post-hoc test, FC ≥ 2, FDR-adjusted $P \leq 0.05$) metabolites in multiple comparisons.

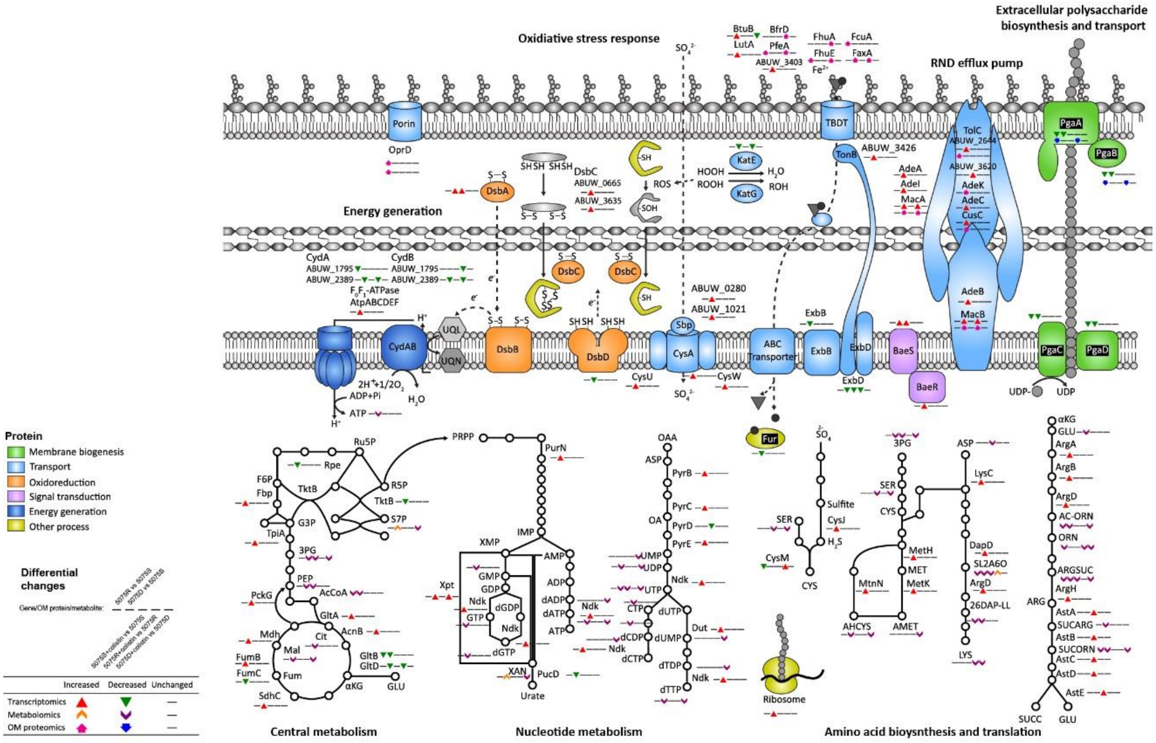

Figure S4. Transcriptomics, metabolomics and OM proteomics analyses showing the significantly perturbed functional pathways apart from membrane biogenesis by either genetic variations or colistin treatments. The genes, metabolites and proteins explicitly referred in text are highlighted with black background. 26DAP-LL, LL-2,6-diaminoheptanedioate; 2PG, 2-phosphoglycerate; 3PG, 3-phosphoglycerate; AcCoA, acetyl-CoA; AC-ORN, *N*^{2}-acetyl-L-ornithine; AHCYS, *S*-adenosyl-L-homocysteine; AMET, *S*-adenosyl-L-methionine; AMP, adenosine monophosphate; ARG, L-arginine; ARGSUC, *N*-(L-arginino)succinate; ASP, L-aspartic acid; Cit, (*R*)-citrate; CMP, cytidine monophosphate; CTP, cytidine triphosphate; CYS, L-cysteine; dADP, deoxyadenosine diphosphate; dATP, deoxyadenosine triphosphate; dCDP, deoxycytidine diphosphate; dCTP, deoxycytidine triphosphate; dGDP, deoxyguanosine diphosphate; dGTP, deoxyguanosine triphosphate; dTDP, thymidine diphosphate; dTTP, thymidine triphosphate; dUDP, deoxyuridine-diphosphate; dUMP, deoxyuridine-monophosphate; dUTP, deoxyuridine-triphosphate; F6P,

D-fructose 6-phosphate; Fbp, D-fructose 1,6-biphosphate; Fum, D-fumurate; G3P, glyceraldehyde 3-phosphate; GDP, guanosine diphosphate; GLU, L-glutaric acid; GLY, glycine; GMP, guanosine monophosphate; GTP, guanosine triphosphate; IMP, inosine monophosphate; LYS, L-lysine; Mal, D-malate; MET, L-methionine; OA, L-orotate; OAA, oxaloacetate; ORN, L-ornithine; PEP, phosphoenolpyruvate; PRPP, phosphoribosyl diphosphate; R5P, D-ribose 5-phosphate; Ru5P, D-ribulose 5-phosphate; S7P, D-sedoheptulose 7-phosphate; SER, L-serine; SL2A6O, *N*-succinyl-2-L-amino-6-oxoheptanedioate; SUCARG, N^2-succinyl-L-arginine; SUCC, succinate; SUCORN, N^2-Succinyl-L-ornithine; UDP, uridine-diphosphate; UMP, uridine-monophosphate; UTP, uridine-triphosphate; XAN, xanthine; XMP, xanthosine monophosphate; αKG, α-ketoglutaric acid.

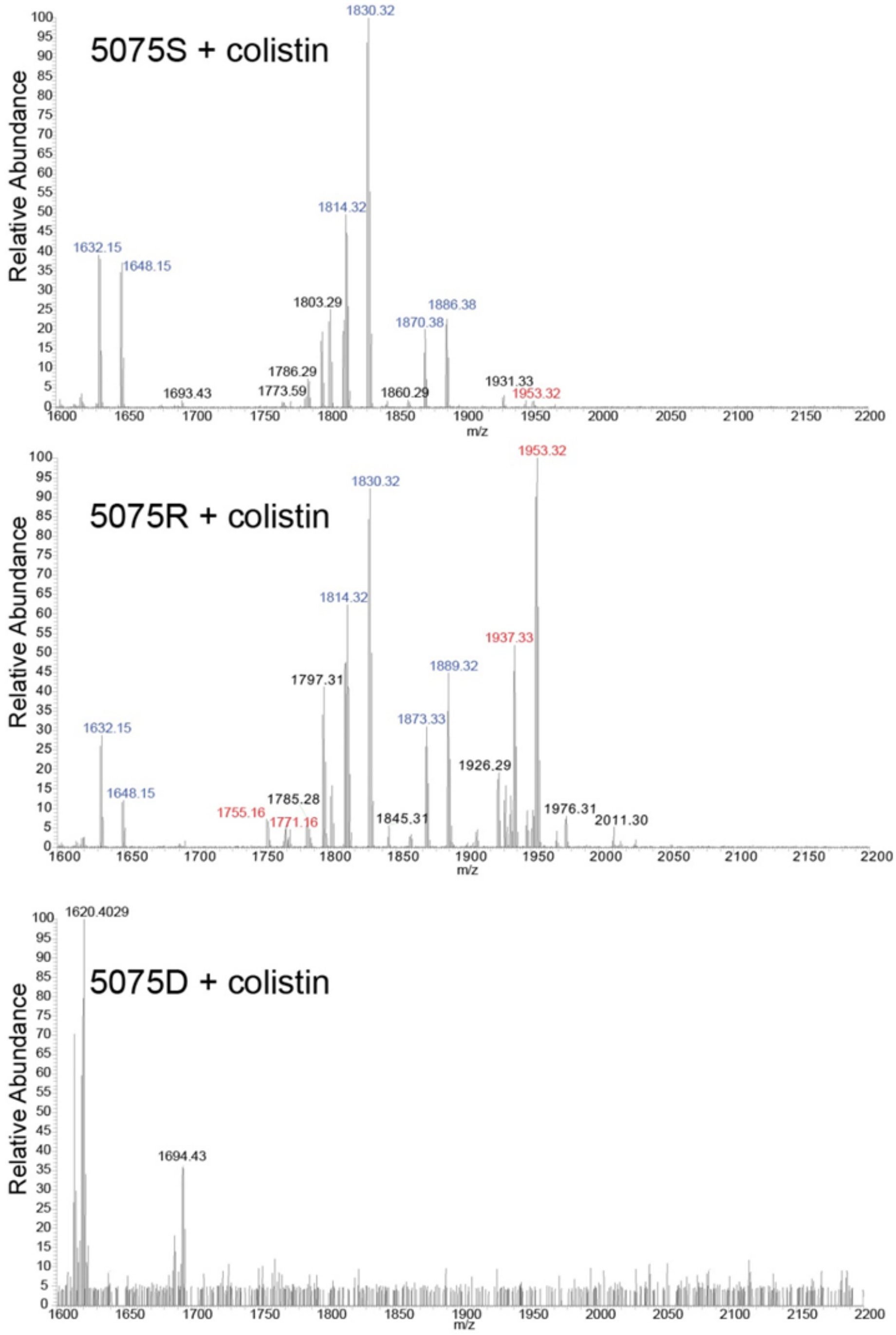

Figure S5. Lipid A profiling of 5075S, 5075R and 5075D after colistin treatment. Samples were collected from the edge of inhibition zones in the disc diffusion assay. The MS peaks representing unmodified and pEtN-modified lipid A are indicated by blue and red, respectively.

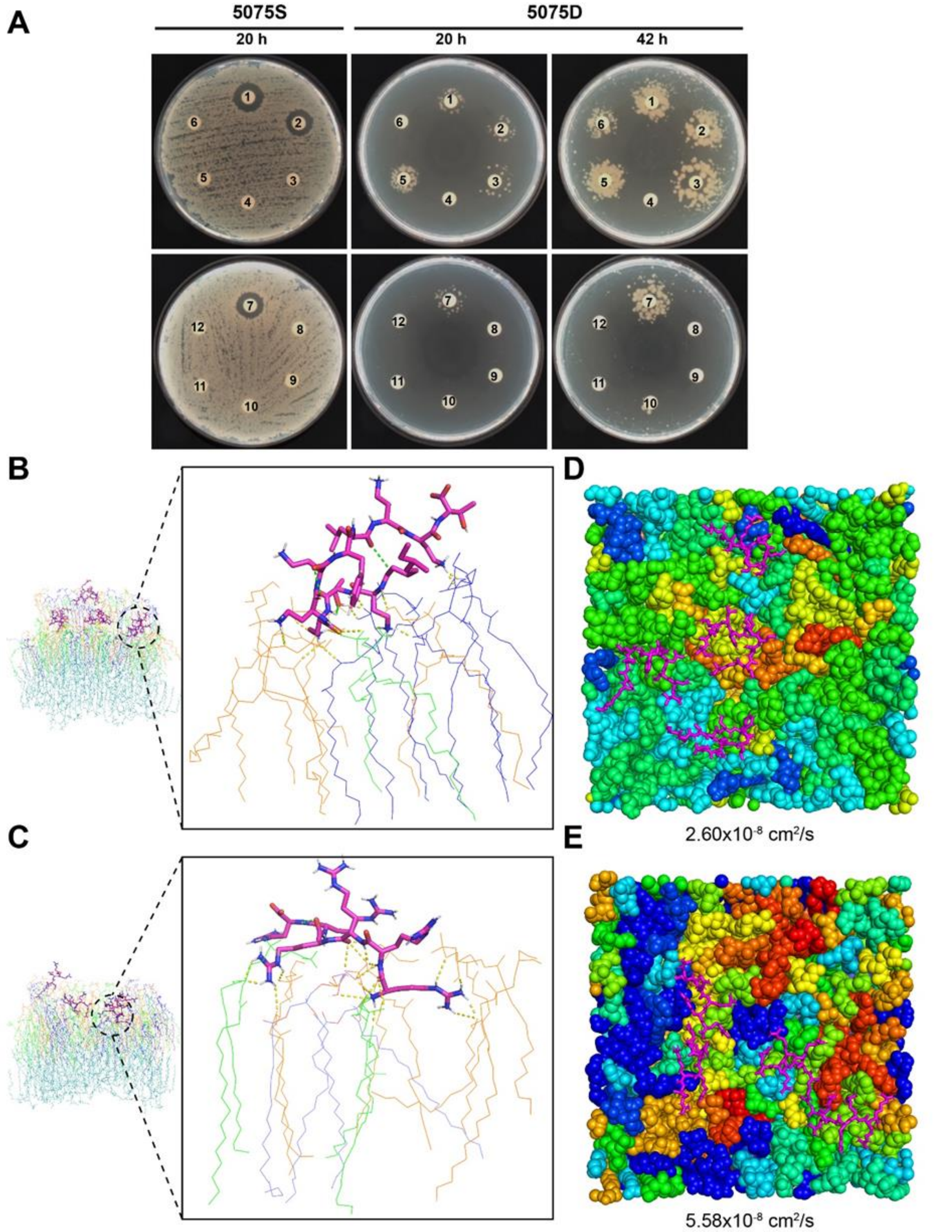

Figure S6. Phenotypic analyses of 5075S and 5075D and molecular dynamics simulations of 5075D OM with polycationic peptides. A) 5075S and 5075D grown on MH agar with discs of (1) and (7) 10-µg colistin, (2) 10-µg polymyxin B, (3) 15-µg linear polymyxin B1, (4) 10-µL 0.9% (w/w) saline, (5) 15-µg polymyxin B1(Lys-1, 3, 5, 8, 9), (6) 15-µg polymyxin B

nonapeptide, (8) 15-μg linear poly-L-arginine, (9) 15-μg linear poly-L-lysine, (10) 50-μg LL-37, (11) 15-μg cyclic poly-L-arginine, and (12) 15-μg cyclic poly-L-lysine**.** B) Molecular interactions between linear polymyxin B (violet sticks) and phospholipid molecules in the outer leaflet of 5075D OM. C) Molecular interactions between linear poly-L-arginine (violet) and phospholipid molecules in the outer leaflet of 5075D OM. The colistin-interacting PG, PE and CL are indicated in orange, green, and dark blue, respectively; while the non-interacting phospholipids in the inner leaflets of OM are indicated in dark teal, respectively. The H-bonds within polycationic peptides and between colistin and phospholipid molecules are indicated by green and yellow dash lines, respectively. D) Binding of linear polymyxin B1 to the OM significantly reduced the overall membrane lateral diffusion coefficient in 5075D. E) Linear poly-L-arginine slightly increased the overall membrane lateral diffusion coefficient in 5075D. Color-coding from blue to red indicates the low to high membrane lateral diffusion coefficient.

Table S1. Minimum inhibitory concentrations (mg/L) of *A. baumannii* strains examined in this study.

Strain	Colistin	Polymyxin B	Rifampicin	Meropenem	Hydrogen peroxide	Ticarcillin	Piperacillin /Tazobactam	Ceftazidime	Ceftriaxone	Cefepime	Amikacin	Gentamicin	Tobramycin	Ciprofloxacin	Trimethoprim /Sulfamethoxazole
5075S	0.25	0.25	2	32	16	≥128	≥128	≥64	≥64	≥64	≥64	≥16	8	≥4	≥320
5075R	16	16		8		≥128	≥128	≥64	≥64	≥64	≥64	≥16	≥16	≥4	≥320
5075D	>128	64	0.125	0.125	4	≤8	≤4	32	32	2	≥64	8	8	≥4	≥320
5075D pWH1266	>128			≤0.25		≤8	≤4	32	32	2	32	8	4	≥4	≥320
5075D pWH1266-*katG*	>128			≤0.25		≤8	≤4	32	32	2	32	8	8	≥4	≥320
5075D pWH1266-*lpxC*	0.25			8		≥128	≥128	≥64	≥64	≥64	≤2	≥16	4	≥4	≥320
AB00379	0.5	0.25													
AB00381	0.25	0.25													
AB00382	0.5	0.5													
AB00796	0.5	0.25													
AB00797	0.5	0.25													
AB00798	1	0.25													
AB09109	0.5	0.5													
AB09110	0.5	0.25													
AB09111	0.5	1													
AB09456	0.5	0.5													
AB09458	0.5	0.5													
AB00256	0.5	0.5													
AB08847	0.5	0.5													

Table S2. Primers, plasmids and strains used in this study.

Primer	Sequence (5'→3')	Note
PmrB_F	ACCTCAATTTTCAGTGTCATCTT	Forward primer for verifying *pmrB* mutation in 5075R
PmrB_R	CACGCTCTTGTTTCATGTAAAT	Reverse primer for verifying *pmrB* mutation in 5075R
0507_F	TGGGAGAATGTAAGCACGCC	Forward primer for verifying ABUW_0507 mutation in 5075R
0507_R	GTTGCTGCTGAGGTGGTGTA	Reverse primer for verifying ABUW_0507 mutation in 5075R
rpoB_F	GTTTCTGTTCGTCATCAAGGT	Forward primer for verifying *rpoB* mutation in 5075R and 5075D
rpoB_R	GTCATCTTTCTCTAAGCCATCAC	Reverse primer for verifying *rpoB* mutation in 5075R and 5075D
0135_F	TGATGAAGAAGGACTCACCA	Forward primer for verifying ABUW_0135 mutation in 5075D
0135_R	TCAGAATAAGAATGCCATGCC	Reverse primer for verifying ABUW_0135 mutation in 5075D
mrcA_F	ACTTATTGCTGAATACGGTGG	Forward primer for verification of *mrcA* mutation in 5075D
mrcA_R	CATAGAGGAATGGTTTGATGGT	Reverse primer for verifying *mrcA* mutation in 5075D
katG_F	AGATGCTGAAGCCATTATTCC	Forward primer for verifying *katG* mutation in 5075D
katG_R	GCCTGATTTACGGTCTTTACCT	Reverse primer for verifying *katG* mutation in 5075D
lpxC_F	GGAATAGGTCTTCATAGCGG	Forward primer for verifying *lpxC* mutation in 5075D
lpxC_R	CATAGCCAAATAAAGACAATGAC	Reverse primer for verifying *lpxC* mutation in 5075D
ISAba1_F	GTGGAATAGCGATACCTATCC	Transposon specific forward primer for verifying IS*Aba1*insertion in *lpxC* in 5075D
katGf	TGCTCCCTGCAGCTCACGGTCTAGCCGATATGG	Forward primer for amplifying *katG* and its native promoter, containing PstI site
katGr	TGCTCCGACGTCTTAAGCTAAGTCAAAACGGT	Reverse primer for amplifying *katG* and its native promoter, containing AatII site
lpxCf	TGCTCCGACGTCACTTTAGCGTACAATCTATCG AAAGGC	Forward primer for amplifying *lpxC* and its native promoter, containing AatII site
lpxCr	TACAAAGAATTCAAGTTCGCTTAAG	Reverse primer for amplifying *lpxC* and its native promoter, containing EcoRI site
pWH1266_F	CTTATCGATGATAAGCTGTCAA	Forward primer for verifying the fragment insertion in pWH1266
pWH1266_R	TAAACAAATAGGGGTTCCGC	Reverse primer for verifying the fragment insertion in pWH1266

Plasmid	Genetic background	Reference
pWH1266	*ori*$_{Ab}$, *tetR*	[4]
pWH1266::*lpxC*	*ori*$_{Ab}$, *tetR*, *lpxC*	This study
pWH1266::*katG*	*ori*$_{Ab}$, *tetR*, *katG*	This study

Strain	Genetic background	Reference
5075S	Wild type	[5]
5075R	*pmrB*^{G315D} (806796G>A), ABUW_0507^{A91P} (517583C>G), and *rpoB*^{F915L} (3652619A>C)	This study
5075D	ABUW_0135^{S25Stop} (148794C>A), *mrcA*^{M129T} (309276A>G), *katG*^{R609G} (3517132C>G), *rpoB*^{F915L} (3652619A>C), and *lpxC*395::IS*AbaI*	This study
5075D pWH1266	Null vector control	This study
5075D pWH1266-*lpxC*	Expression of native *lpxC* in 5075D	This study
5075D *pWH1266-katG*	Expression of native *katG* in 5075D	This study
AB00379	ABUW_0135-46::T26	[6]
AB00381	ABUW_0135-0280::T26	[6]
AB00382	ABUW_0135-399::T26	[6]
AB00796	*mrcA*1925::T26	[6]
AB00797	*mrcA*1268::T26	[6]
AB00798	*mrcA*832::T26	[6]
AB09109	*katG*808::T26	[6]
AB09110	*katG*949::T26	[6]
AB09111	*katG*1900::T26	[6]
AB09456	*rpoB*889::T26	[6]
AB09458	*rpoB*234::T26	[6]
AB00256	*pgpA*::T26	[6]

AB08847	*pgpB*::T26	[6]
ATCC 19606	Wild type	[4]
ATCC 190606R	*lpxA* premature mutation at amino acid position 34	[4]

Table S3. Constructed systems for molecular dynamics simulations.

Strain	System	Outer membrane composition (%)				Number of molecules			
		PE	PG	CL	Lipid A	Polymyxin	Water	Ca^{2+}	Cl^-
5075D	OM	37	47	16	0	0	24,274	78	0
	OM + colistin					4	23,626	68	0
	OM + PMBN					4	23,772	68	0
	OM + linear polymyxin B					4	24,090	68	0
	OM + linear poly-L-arginine					4	24,115	66	0
19606R	OM	64	26	10	0	0	24,079	49	1
	OM + colistin					4	23,409	39	1
PG 20%	OM	65	20	15	0	0	23,363	49	0
	OM + colistin					4	23,482	39	0
PG 30%	OM	55	30	15	0	0	24,181	55	1
	OM + colistin					4	23,569	45	1
PG 35%	OM	50	35	15	0	0	24,200	65	1
	OM + colistin					4	23,585	55	1
PG 40%	OM	45	40	15	0	0	24,262	65	1
	OM + colistin					4	23,614	55	1
PG 50%	OM	35	50	15	0	0	24,342	76	1
	OM + colistin					4	23,688	66	1
5075S	Outer leaflet in OM	18	4	3	75	0	26,387	47	1
	Inner leaflet in OM	72	16	12	0				

Movie S1 (separate file). Molecular dynamics simulation of physical interactions between colistin and 5075D OM in 100 ns. The bacterial OM and colistin molecules are shown as spheres and sticks, respectively. Colistin molecules interacted with the head groups of phospholipids, adhered to the membrane surface steadily, but did not penetrate into the phospholipid bilayer.

Movie S2 (separate file). Molecular dynamics simulation of physical interactions between PMBN and 5075D OM in 100 ns. The bacterial OM and PMBN molecules are shown as spheres and sticks, respectively. PMBN molecules interacted with the head groups of phospholipids, adhered to the membrane surface steadily, but did not penetrate into phospholipid bilayer.

Dataset S1 (separate file). Differentially expressed genes in 5075S, 5075R and 5075D with and without colistin treatment.

Dataset S2 (separate file). Differentially abundant metabolites in 5075S, 5075R and 5075D with and without colistin treatment.

Dataset S3 (separate file). Identified OM proteins in 5075S, 5075R and 5075D with and without colistin treatment.

Dataset S4 (separate file). Phospholipids identified in the OM and IM of 5075S, 5075R, 5075D, 5075D pWH1266-*katG*, 5075D pWH1266-*lpxC*, ATCC 19606 and 19606R.

SI References

[1] M. H. Maifiah, S. E. Cheah, M. D. Johnson, M. L. Han, J. D. Boyce, V. Thamlikitkul, A. Forrest, K. S. Kaye, P. Hertzog, A. W. Purcell, J. Song, T. Velkov, D. J. Creek, J. Li, *Sci. Rep.* **2016**, *6*, 22287.

[2] R. A. Dunstan, E. Heinz, L. C. Wijeyewickrema, R. N. Pike, A. W. Purcell, T. J. Evans, J. Praszkier, R. M. Robins-Browne, R. A. Strugnell, K. V. Korotkov, T. Lithgow, *PLoS Pathog.* **2013**, *9*, e1003117.

[3] R. Jasim, M. L. Han, Y. Zhu, X. Hu, M. H. Hussein, Y. W. Lin, Q. T. Zhou, C. Y. D. Dong, J. Li, T. Velkov, *Int. J. Mol. Sci.* **2018**, *19*, 2356.

[4] J. H. Moffatt, M. Harper, P. Harrison, J. D. Hale, E. Vinogradov, T. Seemann, R. Henry, B. Crane, F. St Michael, A. D. Cox, B. Adler, R. L. Nation, J. Li, J. D. Boyce, *Antimicrob. Agents Chemother.* **2010**, *54*, 4971.

[5] A. C. Jacobs, M. G. Thompson, C. C. Black, J. L. Kessler, L. P. Clark, C. N. McQueary, H. Y. Gancz, B. W. Corey, J. K. Moon, Y. Si, M. T. Owen, J. D. Hallock, Y. I. Kwak, A. Summers, C. Z. Li, D. A. Rasko, W. F. Penwell, C. L. Honnold, M. C. Wise, P. E. Waterman, E. P. Lesho, R. L. Stewart, L. A. Actis, T. J. Palys, D. W. Craft, D. V. Zurawski, *mBio* **2014**, *5*, e01076.

[6] L. A. Gallagher, E. Ramage, E. J. Weiss, M. Radey, H. S. Hayden, K. G. Held, H. K. Huse, D. V. Zurawski, M. J. Brittnacher, C. Manoil, *J. Bacteriol.* **2015**, *197*, 2027.
